# Comparing the cost-effectiveness of the MPT64-antigen detection test to Xpert MTB/RIF and ZN-microscopy for the diagnosis of Extrapulmonary Tuberculosis: An economic evaluation modelling study

**DOI:** 10.1371/journal.pgph.0003414

**Published:** 2024-08-08

**Authors:** Shoaib Hassan, Tehmina Mustafa, William Muller, Lisete Torres, Msafiri Marijani, Esther Ngadaya, Sayoki Mfinanga, Yakobo Lema, Erlend Grønningen, Melissa Jorstad, Ole Norheim, Bjarne Robberstad

**Affiliations:** 1 Department of Global Public Health and Primary Care, Centre for International Health, University of Bergen, Bergen, Norway; 2 Department of Global Public Health and Primary Care, Bergen Centre for Ethics and Priority Setting in Health, University of Bergen, Bergen, Norway; 3 Department of Thoracic Medicine, Haukeland University Hospital, Bergen, Norway; 4 National Institute for Medical Research, Dar es Salaam, The United Republic of Tanzania; 5 The United Republic of Tanzania, Mbeya Zonal Referral Hospital, Mbeya, The United Republic of Tanzania; 6 Mnazi Mmoja Referral Hospital, Zanzibar, The United Republic of Tanzania; 7 Department of Global Public Health and Primary Care, Health Economics, Leadership and Translational Research Group, University of Bergen, Bergen, Norway; Menzies School of Health Research: Charles Darwin University, AUSTRALIA

## Abstract

Extrapulmonary Tuberculosis (EPTB) poses challenges from patient and health system perspectives. The cost-effectiveness analysis of the Xpert MTB/RIF (Xpert) test to diagnose pulmonary tuberculosis is documented. However, there are no economic evaluations for EPTB. Considering the reported better diagnostic sensitivity of the MPT64 test, this study explored its cost-effectiveness as an alternative diagnostic test. We conducted this economic evaluation to assess the cost-effectiveness of the MPT64 test compared to Xpert and ZN microscopy for EPTB adult patients. We utilised a Markov modelling approach to capture short- and long-term costs and benefits from a health system perspective. For the model inputs, we combined data from our cohort studies in Tanzania and peer-reviewed EPTB literature. We calculated the Incremental Cost Effectiveness Ratio (ICER) by comparing the cost (in USD) of each diagnostic test and Quality Adjusted Life Years (QALYs) as health gain. We found the MPT64 test cost-effective for EPTB diagnosis and absolutely dominated ZN microscopy and Xpert using the baseline model inputs. A scenario analysis showed that the Xpert test might be the most cost-effective at its higher test sensitivity, which corresponds to using it to diagnose lymph node aspirates. The prevalence of HIV among EPTB cases, their probability of treatment, costs of ART, and the probability of the MPT64 test in detecting EPTB patients were the main parameters associated with the highest impact on ICER in one-way deterministic analysis. The most cost-effective option for EPTB at the baseline parameters was the MPT64 diagnostic test. Including the MPT64 test in EPTB diagnostic pathways for previously untreated patients can lead to better resource use. The Xpert test was the most cost-effective diagnostic intervention at a higher diagnostic test sensitivity in scenario analyses based on different sites of infection, such as for the lymph node aspirates.

## Background

Extrapulmonary Tuberculosis (EPTB) patients currently represent 15–24% of the 10 million tuberculosis (TB) patients reported globally [1]. Although there has been a reduction in the overall prevalence of TB, a similar decrease in the proportion of EPTB disease burden is not observed [2]. The reasons for this relatively lower decline in the EPTB disease burden are not clearly demarcated. Surveillance data suggest that, after adjustment for age, sex, and TB treatment history, TB cases from India, Africa, and Asia were more likely to develop EPTB than those from Europe or the Americas [3]. The World Health Organization (WHO) published the 2020 End TB Strategy, which includes till 2025 achieving the milestones to push the annual decline in TB incidence and reducing TB-associated mortality by 10% and 5%, respectively, compared to 2015 [2]. Unless the EPTB disease burden is addressed, it will be challenging to achieve these targets. The latest TB report highlighted coinfections among TB patients. Among other risk factors for acquiring TB, the report highlighted that the number of TB infections could be reduced by addressing HIV coinfections [2]. A delayed diagnosis of ETPB-HIV coinfected cases has led to increased morbidity and mortality. The disease outcome for patients may be better if an earlier identification and treatment are available [4]. However, timely and accurate EPTB diagnosis is an ongoing global challenge [5–8]. Several laboratory tests are commonly used for diagnostic confirmation of TB patients, but Xpert MTB/RIF assay (Xpert) and Ziehl-Neelsen (ZN) microscopy are the most common practice [6, 9–11]. Although the WHO has approved the Xpert test performed using the GeneXpert platform and recommended its implementation for PTB and EPTB diagnosis, it is not without limitations [1, 12–14].

Evidence suggests that Xpert has a lower diagnostic sensitivity for EPTB than a composite reference standard (CRS) [13, 15]. The diagnostic sensitivity of ZN microscopy for EPTB diagnosis is also far from optimal [6, 15]. An alternative is a newly developed immunohistochemistry-based test, the MPT64 antigen detection (MPT64) test, that showed higher diagnostic sensitivity compared to Xpert and ZN microscopy for the diagnostic confirmation of EPTB cases alone as well as HIV coinfections [15–18]. The test was developed and implemented as part of a project to improve EPTB diagnosis by antigen detection from extrapulmonary samples by immunochemistry-based assays. This project included implementing multicentered prospective cohort studies of EPTB patients in the United Republic of Tanzania and India. Implementation of this test could contribute towards timely and accurate diagnosis and treatment.

The use and cost-effectiveness of Xpert and ZN microscopy to diagnose pulmonary TB is well established, but there has been no similar economic evaluation of these tests for EPTB [11, 19–33]. Furthermore, the newly proposed better MPT64 test for diagnosing EPTB warrants a cost-effectiveness analysis compared to the other two diagnostic methods. Additionally, this study is the first economic evaluation that employs Quality-Adjusted Life Years (QALYs) as a payoff for EPTB patients. Here, we report the economic evaluation modelling study results that compare the cost-effectiveness of three EPTB diagnostic tests, including the Xpert, ZN microscopy (as current practice) and MPT64 test.

## Methods

### Model overview

We developed a decision model based on cohort studies in Tanzania to compare the cost-effectiveness of the above-mentioned three laboratory methods of diagnosing EPTB. We established the model using TreeAge Pro software version 2023 (TreeAge, Williamstown, Massachusetts) and reported results following the Consolidated Health Economic Evaluation Reporting Standards (CHEERS) guideline [34]. We estimated health effects as QALYs and costs in USD. Our model had a cycle length of one year and ran for 60 cycles until all individuals of the study cohort (mean starting age 40 years) ended up in the absorbing states (recovered or died). The cycle length of one year is in line with the usual complete duration of EPTB treatment (within 12 months) and the estimation of annual gain in health utilities. This model also considered HIV co-infections among EPTB patients. However, it assumed good compliance with antiretroviral treatment (ART) that did not vary regardless of using any of the three EPTB diagnostic tests.

We considered costs from the healthcare providers’ perspective, as all eligible TB patients should receive the diagnostic interventions and treatment free of payment (6). However, TB patients may also bear indirect costs in accessing healthcare, especially in the low-middle income setting, which should also be studied in future from a patient’s perspective. We used a 3% discount rate for both costs and QALYs in our modelling study [35]. Results are reported as the Incremental cost-effectiveness ratios (ICERs) and presented by Cost Effectiveness Acceptability Curve (CEAC) that showed the model iteration presenting a cost-effective option compared to others at a certain willingness to pay (WTP) as the USD per QALY. The WTP is the maximum monetary spending for a unit gain in utility. For our study, we arbitrarily choose to use the upper limit of a cost-effectiveness threshold estimated by Woods *et al*., whose methodology and details are available elsewhere [36]. The three diagnostic tests under economic evaluation in this model were also plotted in a pairwise comparison of incremental cost and effectiveness presented by the incremental cost-effectiveness scatterplot. The patients and public were not involved in any way in this economic evaluation model.

We used a decision analytical tree to capture the short-term diagnostic pathways. The diagnostic outcomes were modelled in a Markov model, which captured long-term costs and consequences. The utilisation of treatment depended on the diagnostic confirmation of EPTB and HIV, with subsequent implications over a lifetime horizon. This study modelled EPTB patients who had not received antituberculosis treatment (ATT) during the previous year.

### Decision tree model for short-term outcomes

**Fig 1:**
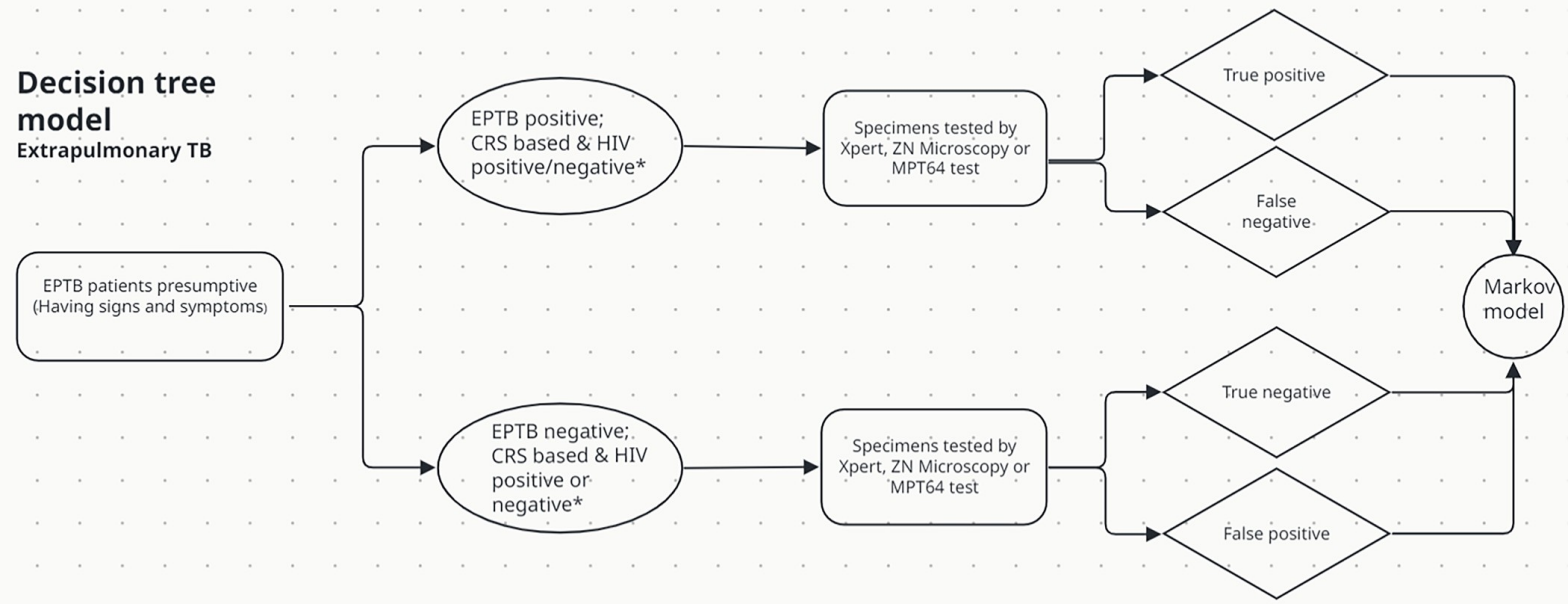
Pathway illustrating a simplified flow of EPTB patients (including HIV co-infection), and their subgroups based on diagnostic tests’ accuracies against the CRS. *Following EPTB case classification (based on CRS), patients got HIV diagnostics and are categorised into four sub-groups.

We initiated the model structure with the cohort of patients having presumptive signs and symptoms of EPTB illness entering the decision tree (Fig 1). Firstly, patients were categorised as EPTB positive or negative according to a CRS criterion and then received results of HIV diagnostic confirmation [7, 15]. These two steps led to four sub-groups of cases: (i) EPTB positive and HIV positive, (ii) EPTB positive and HIV negative, (iii) EPTB negative and HIV positive, and (iv) EPTB negative and HIV negative. The inclusion of CRS was adapted from our cohort study in Tanzania. It was used from a modelling perspective only to estimate diagnostic accuracies (Table 1) and had no implication in clinical practice. Secondly, to obtain laboratory confirmation of EPTB, one specimen per patient was modelled to be separately tested by three diagnostic tests under economic evaluation: the Xpert, ZN microscopy, and MPT64 test. Using the same specimen from each EPTB patient would prevent diagnostic accuracies being influenced by variations in the specimen collection or handling. Furthermore, the enclosure of diagnostic tests’ accuracies allowed labelling of the cohort into additional subgroups such as (i) true positive, (ii) false negative, (iii) true negative and (iv) false positive (simplified illustration shown in Fig 1).

**Table 1:** Model input parameters include probabilities of diagnostic tests’ accuracies and treatment success by subgroups.

Parameter	Distributions	Probability (SE)	Reference
Confirmed EPTB based on CRS among patients having presumptive signs and symptoms	Beta	0.54 (0.03)	[7, 15, 17]
			Own data
HIV +ve among EPTB positive patients	Beta	0.35 (0.03)	[7, 15, 17]
			Own data
HIV +ve among EPTB negative patients	Beta	0.31 (0.04)	[7, 15, 17]
			Own data
Sensitivity of ZN microscopy among HIV +ve	Beta	0.21 (0.11)	[7, 15, 17]
			Own data
Sensitivity of ZN microscopy among HIV -ve	Beta	0.11 (0.06)	[7, 15, 17]
			Own data
Specificity of ZN microscopy among HIV +ve	Beta	0.99 (0.01)	[7, 15, 17]
			Own data
Specificity of ZN microscopy among HIV -ve	Beta	0.99 (0.01)	[7, 15, 17]
			Own data
Sensitivity of Xpert among HIV +ve	Beta	0.13 (0.07)	[7, 15, 17]
			Own data
Sensitivity of Xpert among HIV -ve	Beta	0.27 (0.07)	[7, 15, 17]
			Own data
Specificity of Xpert among HIV +ve	Beta	0.99 (0.01)	[7, 15, 17]
			Own data
Specificity of Xpert among HIV -ve	Beta	0.99 (0.01)	[7, 15, 17]
			Own data
Sensitivity of MPT64 among HIV +ve	Beta	0.70 (0.08)	[7, 15, 17]
			Own data
Sensitivity of MPT64 among HIV -ve	Beta	0.54 (0.10)	[7, 15, 17]
			Own data
Specificity of MPT64 among HIV +ve	Beta	0.89 (0.07)	[7, 15, 17]
			Own data
Specificity of MPT64 among HIV -ve	Beta	0.94 (0.06)	[7, 15, 17]
			Own data
Completion of treatment among TB positive HIV positive	Beta	0.83 (0.05)	[40, 51]
Completion of treatment among TB positive HIV negative	Beta	0.89 (0.05)	[37, 38]
			Own data
Completion of treatment among TB negative HIV positive	Beta	0.88 (0.05)	[52]
False negative classified patients receiving treatment elsewhere	Beta	0.33 (0.05)	Model assumption
Mortality among DST EPTB positive HIV negative patients on treatment^¤^	Beta	0.07 (0.05)	[50]
Mortality among DST EPTB positive HIV negative patients not on treatment^¤^	Beta	0.09 (0.05)	Model assumption
Mortality among DST EPTB positive HIV positive patients on treatment^¤^	Beta	0.14 (0.05)	[50]
Mortality among DST EPTB positive HIV positive patients not on treatment^¤^	Beta	0.15 (0.05)	Model assumption
Mortality among MDR EPTB positive HIV negative patients on treatment^#^	Beta	0.16 (0.05)	Model assumption
Mortality among MDR EPTB positive HIV negative patients not on treatment^§^	Beta	0.18 (0.05)	Model assumption
Mortality among MDR EPTB positive HIV positive patients on treatment^#^	Beta	0.19 (0.05)	Model assumption
Mortality among MDR EPTB positive HIV positive patients not on treatment^§^	Beta	0.20 (0.05)	Model assumption
Treatment failure/transferred/not registered	Beta	0.045 (0.009)	[37] SE are model assumption
Treatment completion among failure/transferred/not registered	Beta	0.066 (0.009)	[53] SE are model assumption
Loss to follow up (LTFU)	Beta	0.038 (0.009)	[37] SE are model assumption
Retreatment of LTFU	Beta	0.066 (0.009)	[53] SE are model assumption

*Standard error

¤ Using either of the three tests under this economic evaluation.

# Using the Xpert test.

§ Using the ZN microscopy and MPT64 tests.

### Markov model for long-term outcomes

Following the known diagnostic process and exhaustive outcome flow of EPTB patients based on a multicentered study, the Markov life cycle model captured long-term outcomes [37]. The Markov model had four distinct states for EPTB patients at the time of diagnosis: (i) drug-sensitive treatment (DST), (ii) multi-drug resistant (MDR) treatment, (iii) recovered and (iv) death. EPTB has a low MDR and mortality associated with it, but without modelling these states, the model would not be exhaustive. After the diagnostic process, EPTB patients mainly receive DST, which can have several outcomes as modelled as reported per a multicenter study.

EPTB patients grouped in the true positive arm were modelled to have Markov states like continuing treatment to successful completion, died, loss to follow up (LTFU), and treatment failure, transferred or not registered. Based on a retreatment rate (reported per available literature) among TB patients that included EPTB cases too, LTFU patients at a certain probability (Table 1) were modelled to continue EPTB treatment elsewhere (see Figs A-H in S1 Text). The LTFU and treatment failure arms were also modelled to have similar Markov states like the ones in alive and completed treatment pathway. Noteworthy that only the Xpert test had the ability to detect MDR EPTB patients that could receive appropriate treatment and was modelled accordingly (see Figs A-H in S1 Text). The use of ZN microscopy and MPT64 test did not identify the MDR status of EPTB cases. Hence, these patients would not have received treatment appropriate for their MDR status. Patients in the false negative arm would usually not receive EPTB treatment. However, for the false negative arm of EPTB patients, we assumed that a third of these patients may also receive EPTB treatment elsewhere for this illness and were modelled accordingly (see Figs E-H in S1 Text). The payoffs for patients in this group who were treated for MDR (after being diagnosed by using the Xpert test) and receiving treatment elsewhere were modelled like a successful treatment like the ones in the true positive group. No additional drug sensitivity tests were modelled.

During the 60 annual cycles, the cohort gradually ended up in the absorbing state of “recovered” or “died” [7, 15, 38–42]. The recovered state (with an initial probability <0.001) was set up to capture those who successfully complete EPTB treatment and live with a background population mortality rate based on WHO’s estimated life expectancies for the Republic of Tanzania [43]. At the end of each annual cycle, our model captured QALYs and costs as stage rewards, and patients could remain in their current live state or die. The model did not consider relapse or reinfection for successfully treated patients as it would be a different healthcare pathway depicting EPTB patients with disease-associated relapses and complications. These patients might have received EPTB treatment in the last 12 months and were beyond the inclusion criteria of our cohort studies whose data is employed in the model. HIV-positive patients were modelled to continue receiving ARTs for a lifespan [44]. They were not penalised for additional risk and were modelled to have mortality and morbidity as the healthy population.

### Data sources for parameters

Most of the values for the model variables came from our prospective cohort studies in Tanzania and peer-reviewed literature, as referenced in this manuscript. Our cohort studies were conducted in Zanzibar and Mbeya districts of Tanzania from 1 August 2014 to 31 August 2015 and 1 April 2016 to 31 July 2017, respectively. For the current economic evaluation, we used data for EPTB and HIV diagnostic results of adult patients from these two study sites (Table 1). For the patient-reported health profiles, we had EQ-5D-3L data both before as well as after the EPTB diagnostic and treatment intervention from the Zanzibar study site (Table 2).

**Table 2:** Mean values for the health utilities for EPTB positive patients by HIV positive and negative subgroups (mean age 40 years).

Utilities	Distributions	Mean*	SDs*	Reference
**Utilities based on case-based EQ-5D-3L Health Profiles**				Own data, [7], in S1 Text
EPTB positive and HIV negative cases before treatment (n = 26)	Beta	0.73	0.22	Own data, [7], in S1 Text
EPTB positive and HIV positive cases before treatment (n = 5)	Beta	0.58	0.22	Own data, [7], in S1 Text
EPTB positive and HIV negative cases after treatment (n = 26)	Beta	0.97	0.05	Own data, [7], in S1 Text
EPTB positive and HIV positive cases after treatment (n = 5)	Beta	0.9	0.01	Own data, [7], in S1 Text
EPTB negative and HIV positive cases after treatment	Beta	0.94	0.05	[56]
EPTB negative HIV positive patients when treatment not received¤	Beta	0.58	0.22	Own data, [7], in S1 Text
EPTB negative HIV negative patients when treatment not received¤	Beta	0.73	0.22	Own data, [7], in S1 Text
Health utility assumed when treatment not required (EPTB negative HIV negative)	Beta	0.99	0.01	Own data, [7], in S1 Text

*Mean and Standard Deviations allow estimation of Alpha and Beta parameters, a built-in function of the TreeAge software

¤ Corresponds to the EPTB patients when treatment is not received.

The probability of an individual receiving EPTB case confirmation was based on the diagnostic tests’ accuracies per our cohort studies in Tanzania. The diagnostic tests’ accuracies were internally validated against a CRS whose details are published elsewhere (see Table 1 for parameter estimates) [7, 15, 17]. Since the Xpert test was under economic evaluation, we did not include it as part of the CRS. Our cohort study did not aim at follow-up of HIV patients. Therefore, we modelled adherence to ART among asymptomatic HIV patients based on the published literature (Table 1). The cost of the MPT64 test was based on immunohistochemistry diagnostics offered in private-sector laboratories, which also matched our internal costing estimates [45]. The cost of Xpert, ZN microscopy, HIV test, ART and ATT were obtained from peer-reviewed literature (Table 3) [46–49].

**Table 3:** Mean values for the cost parameters and inputs for the probabilistic sensitivity analysis.

	Distributions	Mean*	SDs*	Reference
Cost of Xpert test	Gamma	29	3	[48]
Cost of MPT64 test	Gamma	31	3	[45]
Cost of ZN microscopy	Gamma	14	7	[47, 57]
Cost of HIV test	Gamma	33	3	[47, 57]
Cost of first-line TB treatment	Gamma	144	71	[47, 57]
Cost of second-line TB treatment	Gamma	3457	1069	[47, 57]
Cost of HIV treatment	Gamma	404	110	[48]
Cost of HIV treatment in loss-to-follow up patients	Gamma	163	58	[48]

*Mean and Standard Deviations allow estimation of Alpha and Lambda parameters, a built-in function of the TreeAge software

The probabilities of successful completion of treatment were retrieved from peer-reviewed literature [37, 50]. The mortality rates of EPTB cases on treatment by HIV status are not widely available. However, we could retrieve a peer-reviewed study from Benin to model mortality rates. Patients not receiving appropriate EPTB treatment (DST or MDR) are not followed up in healthcare pathways, and their mortality rate is not reported in the literature. Therefore, we assumed the mortality rate was higher than the ones on appropriate EPTB treatment in their respective categories (Table 1). The long-term (beyond one year) background mortality of patients (that either recovered following the successfully completion of treatment, were false positive or true negative EPTB cases) were modelled according to the WHO’s estimated life expectancies for the Republic of Tanzania [43].

All three diagnostic tests had a high specificity (Table 1). However, for modelling purposes, we included false positive (FP) patients being offered treatment due to the presence of signs and symptoms. There is no published data to model this scenario; therefore, we used the treatment compliance rate among the FP patients as with the true positive EPTB patients. Noteworthy that while EPTB treatment of false positive patients would have a financial implication for the health system, it would not necessarily result in additional health utilities as in principle, EPTB treatment is not required for these patients. Other than patients falsely labelled EPTB positive (false positive), EPTB and HIV negative patients were not modelled to receive any treatment due to the mere presence of signs and symptoms mimicking EPTB (see Figs A-L in S1 Text).

### Health utilities

The utilities for QALYs estimates in our model were based on the already published prospectively collected EQ-5D-3L profiles of EPTB patients [7, 54]. To estimate these utilities, we used the EQ-5D-3L value set from Zimbabwe, the only country in the region for whom there are value sets available to estimate health utilities while using the EQ-5D-3L profiles [55]. At the end of each cycle, our model estimated QALYs of patients’ sub-groups by EPTB-HIV comorbidity status (Table 2). We assumed that patients correctly testing negative for both EPTB and HIV in the model would not require either EPTB or HIV treatment. The utilities of these patients were assumed to be closer to the healthy population (Table 2). However, due to the presence of signs and symptoms, their utilities were assumed to be at 0.99, slightly below the maximum value of 1.0. Their reported signs and symptoms may be due to illnesses other than EPTB. Such patients were suggested to follow up for additional diagnostic workup and may have received alternate diagnoses for non-EPTB patients not included in this economic evaluation study [15, 18]. The health utility of false negative patients during the first model cycle was sub-grouped based on their HIV status, either positive or negative (Table 2). Without available literature, we assumed that two-thirds of these patients would not receive treatment, and their health utilities would decrease by one percentage point every second year.

The MDR false negative cases were modelled with an additional decrement of 1% every second year. By this step, we modelled utilities of false negative patients who missed the opportunity of EPTB treatment. However, if patients were erroneously labelled as EPTB positive (false positive), their utilities for the first year of treatment were assumed to be equal to the mean utility value of the entire EPTP cohort (0.71). This approach helped model approximate side effects or complications of the unwarranted treatment. No long-term (beyond one year) side effects of unwanted treatment were assumed, and for the subsequent years, utilities of the false positive patients were assumed like other non-EPTB patients. Following treatment, the health utilities of HIV positive patients were assumed to be the same as for the healthy population (1). A summary of health utilities estimation and corresponding input values for our model can be found in Table 2 and Tables A-B in S1 Text.

### Cost data

The cost parameters included in our model were based on reported extracts from health services providers’ perspectives. We collated cost parameters from within Tanzania, where our cohort study was based or from similar settings in neighbouring countries (referenced in Table 3).

We retrieved the cost estimates of Xpert test and HIV treatment from a peer-reviewed Tanzanian study, which used micro-costing from the providers’ perspective. The estimates included recurrent (e.g., personnel time, material and supplies, electricity, and transportation) and capital (e.g., building, equipment, and transport vehicles) costs. The capital costs were annualised using a discount rate of 7.8% with an expected useful life of 3–15 years for equipment and three years for training of lay workers [48]. The Xpert test in approximately 145 countries has been accessible at a 45% price reduction due to either procurement at subsided cost or supplies from donor agencies [12]. While Xpert’s economic (non-subsided) cost may be higher than the currently incurred financial (subsided) costs from providers’ perspective, especially in countries with a high TB prevalence, it did not represent the actual cost from a health system perspective. Therefore, we used the cost parameters that were representative of its utilisation (subsided cost of Xpert test from a health system perspective) in the East African and Indian settings, where our multi-centred cohort studies were based (Table 3) [30].

Other important model inputs were costs of HIV test and EPTB treatment that we retrieved from a multicentered international study that followed Costing Guidelines for Tuberculosis Interventions and Value TB protocol [47, 57]. The costs were estimated using a bottom-up approach from healthcare providers’ perspectives in Kenya and reported disaggregated number of units (measurement) and their values. Like other costs used in our model, these too included recurrent (personnel time, medical supplies) and capital costs that were annuitised at a 3% discount rate. A more detailed description of the costing methodology and resulting estimates are part of a larger database [47, 57]. The HIV test cost was representative of existing diagnostic practices and included the costs of outpatient triage, HIV rapid and confirmatory tests, screening, and treatment visits [57].

Additional interventions could often be employed to obtain a biological specimen for laboratory confirmation of EPTB, such as an X-ray, ultrasound, and other diagnostic tests or clinical workup. We modelled the use of biological material (taken from the site of infection for a patient) for the same specimen to be tested by all three diagnostic tests. Therefore, the costs of additional interventions were not required as a model input since they would not affect incremental costs among the tests under economic evaluation in this study. The cost of EPTB and HIV diagnostic tests was incurred only one time. The cost of treatment for EPTB and HIV positive patients was included at the start of the model following the standard treatment. Moreover, the ART cost was also included at the start of the model for HIV positive patients. Our model also accounted for an initial one-time cost for the diagnostic workup and treatment of HIV positive patients that either died or were lost from follow-up within three months of the first encounter.

We converted all costs equivalent to USD using 2021 as the base year [58]. To cater to any variation in real-life scenarios, we performed probability sensitivity analyses (PSA) based on the values of the model parameters (Table 3 and Table C in S1 Text). The input of the modelled cost parameters would vary based on the diagnostic tests’ accuracies for EPTB (see Figs A-L in S1 Text).

### Sensitivity analyses

Uncertainty analyses were performed in two steps. Firstly, a one-way deterministic sensitivity analysis (DSA) was conducted to explore how realistic ranges for the individual parameters might have affected the ICER. We used a tornado diagram to present these results, while the parameters with the most impact on estimated ICER are shown on top. For the DSA, the parameters’ minimum and maximum values were defined by their standard deviations. In the absence of information about the standard deviation of the diagnostic tests’ costs, we varied minimum and maximum values by +/- 30% around the mean values for the DSA.

Secondly, we performed probabilistic sensitivity analysis (PSA) to assess the simultaneous uncertainty in all the model parameters [59]. The process involved a Monte Carlo simulation for 10,000 iterations with random draws of parameter values from their assigned distributions (Tables 1–3). For the PSA, the diagnostic tests’ accuracies and utility parameters were modelled as beta distributions, having values restricted by the range 0–1. Considering a large variation in the Xpert test’s diagnostic accuracy based on the body site infected, we conducted additional scenario analyses. For this purpose, we also adopted a higher probability of test sensitivity around 0.49 (SE = 0.05) and 0.79 (SE = 0.05) for the Xpert test to diagnose EPTB, especially in biological samples from lymph node fluid aspirates as reported in a systematic literature review [60]. We used Gamma distributions for cost variables, which are non-negative and positively skewed by nature [61]. The mean values of the model parameters are reported in Tables 1–3. The upper and lower ranges of diagnostic tests’ costs and all parameters for the probability of successful treatment completion were unavailable from our cohort studies; therefore, for these variables, we adopted standard deviations from peer-reviewed literature (Tables 1 & 3). The results of the PSA are reported as a cost-effectiveness acceptability curve and cost-effectiveness scatter plot.

To assess the cost-effectiveness of three diagnostic strategies, we defined our WTP threshold at the commonly adopted 1.5 times the Gross Domestic Product (GDP) per capita. Therefore, our defined WTP threshold for the base case model was USD 1790 (1.5 times the Tanzanian GDP per capita of 1193 USD) [62]. We also analysed the cost-effectiveness for the sensitivity analyses at the arbitrarily higher WTP threshold of USD 3579, three times the GDP per capita.

### Ethical considerations

For the prospective cohort study, ethical clearance was obtained from the Regional Committee for Medical and Health Research Ethics, Western-Norway (REK Vest) 2014/46/REK vest, the Zanzibar Medical Research and Ethics Committee (ZAMREC) ZAMREC/0001/MAY/014 and the Ethical committee for biomedical research at the National Medical Research Coordinating Committee in Tanzania NIMR/HQ/R.8a/Vol.IX/2142. All study participants provided informed written consent.

### Role of the funding source

The Research Council of Norway’s funding supported the original cohort study, whose data is utilised in the economic evaluation. No funding for this economic evaluation has been received, including study design, data collection, data analysis, data interpretation, or report writing. The corresponding author had full access to all the data in the study and was final responsible for the decision to submit it for publication. One of the co-authors (TM) was the principal lead in developing the MPT64 test.

## Results

The model estimated that using the MPT64 test as a diagnostic intervention would cost less over the life cycle of patients and result in greater benefits compared to using Xpert and ZN microscopy. The model output (per patient) showed that at the above-mentioned baseline parameters, the MPT64 test absolutely dominated ZN microscopy and Xpert test. The MPT64 arm of diagnostic intervention among the EPTB-HIV coinfected population would cost USD 1210 and yield 12.89 QALYs (Fig M in S1 Text). The cost-effectiveness acceptability curve illustrated that 100% of the model iterations showed the MPT64 test the most cost-effective option per QALY gain at three times the GDP per capita for Tanzania (Fig N in S1 Text).

### Sensitivity analyses

Using the Xpert test sensitivity of 0.49 (SE = 0.05) as part of the sensitivity analyses, the use of the Xpert test and ZN microscopy was absolutely dominated by the MPT64 test. We found that at our defined WTP threshold, MPT64 and Xpert test were most effective in above 98% and 2% of the model iterations, respectively. Once we used the WTP threshold as three times the GDP per capita for Tanzania, MPT64 and Xpert were most cost-effective in 95% and 5% of the model iterations. The ZN microscopy use was not cost-effective in either of these sensitivity analysis scenarios.

We also employed the higher diagnostic sensitivity of the Xpert test at 0.79 (SE = 0.05) in our sensitivity analyses. Per these model inputs, we estimated that using the Xpert test as a diagnostic intervention would cost more over the life cycle of patients but result in higher benefits compared to using MPT64 and ZN microscopy (Table 4). The resultant output showed that at our defined WTP, the Xpert test and MPT 64 were cost-effective in 77% and 23% of the model iterations, respectively. At the lower WTP thresholds (until below USD 1056), about 51% of the model iterations showed that MPT64 was the most cost-effective test, followed by the Xpert test in 49% of model iterations. Above this WTP, the most cost-effective option to diagnose EPTB alternated to Xpert. All the model iterations were plotted on the cost-effectiveness plane as we ran the Monte Carlo simulations (Fig O in S1 Text). If the WTP was even higher, at the threshold of three times the GDP per capita, about 90% of the model iterations would show the Xpert test as the most cost-effective diagnostic test, followed by MPT64 (10%).

**Table 4:** Incremental cost and effectiveness comparing Xpert and MPT64 tests (excluding the absolutely dominated ZN microscopy) to diagnose EPTB patients (model output resulting from 10,000 Monte Carlo simulations).

	Cost (USD, 95% Upper- Lower bound)	Incremental Cost (USD)	Effectiveness (QALYs, 95% Upper- Lower bound)	Incremental effectiveness (QALY)	ICER (USD/QALY)
**MPT64**	1210 (1200–1219)		12.90 (12.81–12.98)		
**Xpert**	1947 (1931–1962)	737 (731–744)	13.61 (13.52–13.69)	0.71 (0.70–0.72)	1038

**Fig 2:**
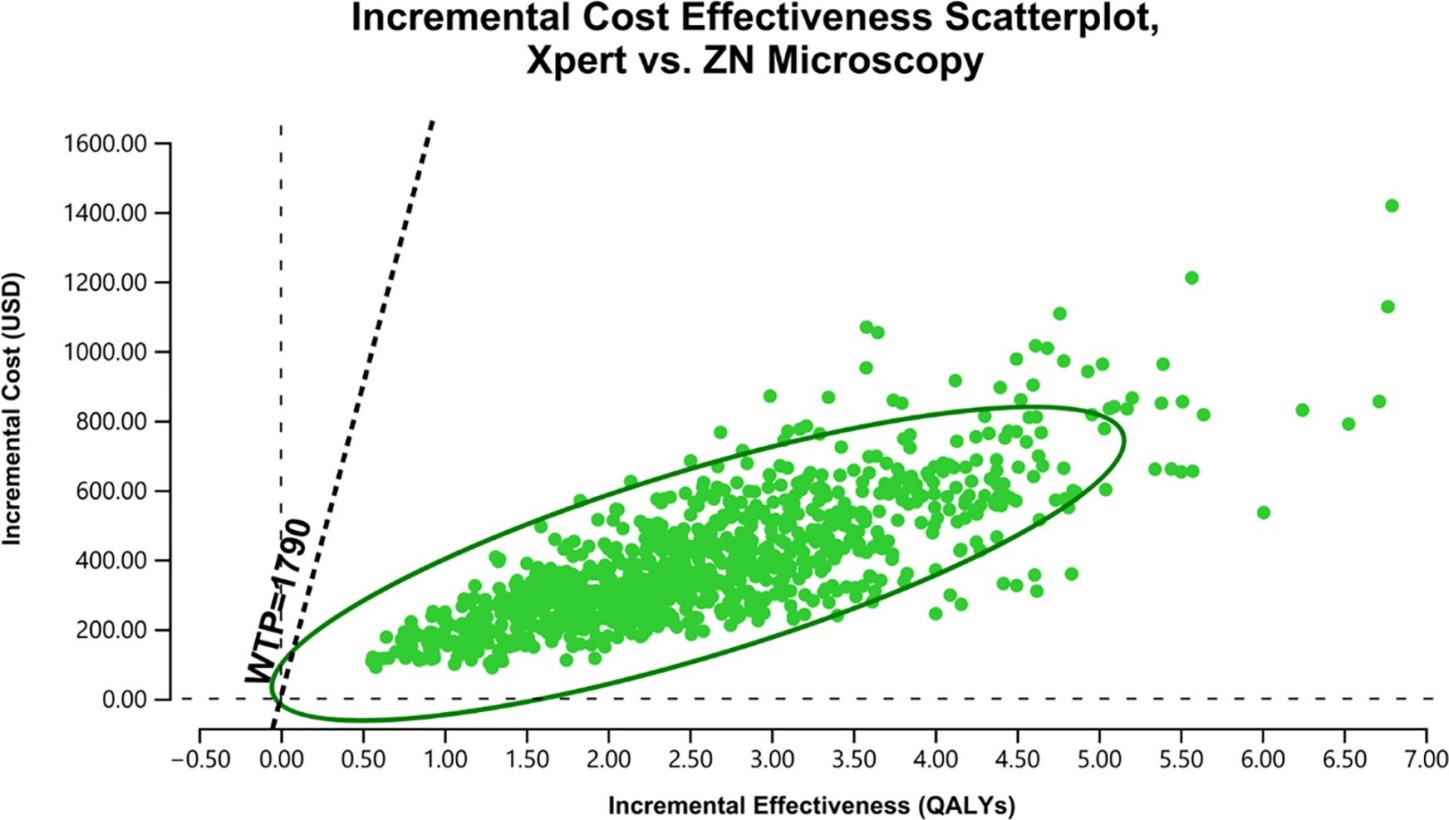
ICE Scatterplot, Xpert vs ZN microscopy.

**Fig 3:**
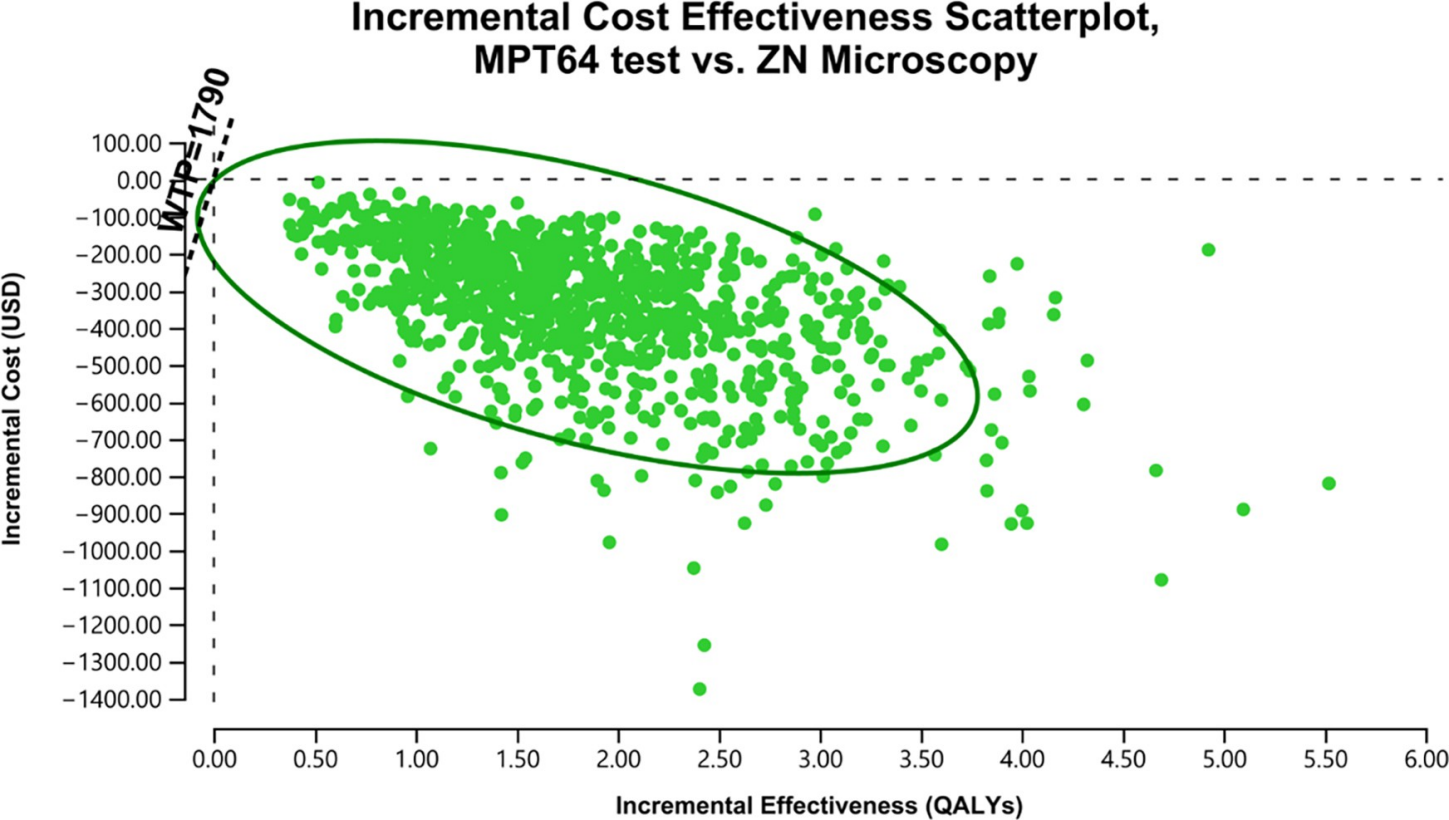
ICE Scatterplot, MPT64 vs ZN microscopy.

**Fig 4:**
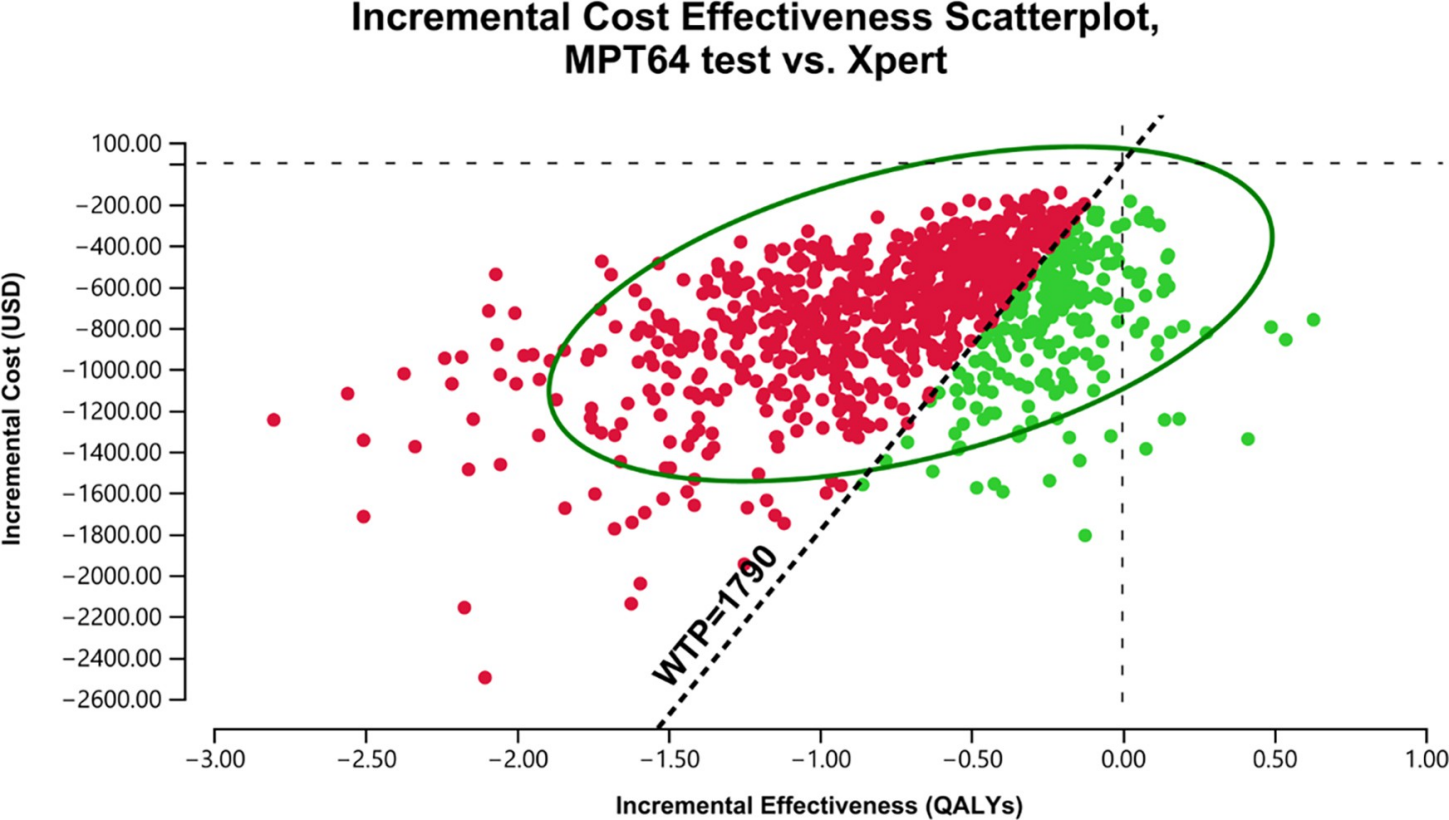
ICE Scatterplot, MPT64 vs Xpert. The ellipse presents a 95% confidence interval, and the diagonal dotted line is a WTP threshold of USD 1790. Markov model Monte Carlo iterations (for a PSA scenario at Xpert test sensitivity of 0.79) show incremental cost-effectiveness (ICE) estimation in a pairwise comparison of the three diagnostic tests under economic evaluation. The red and green colours show corresponding points per labelled axis above and below the WTP threshold (WTP) of 1790 USD, respectively. Horizontal and vertical dotted lines denote the four quadrants of a cost-effectiveness plane.

Per model iterations, at the Xpert’s sensitivity of 0.79 (SE = 0.05), the Xpert test was more cost-effective for 99% of iterations compared to the ZN microscopy. (Fig 2). The MPT64 test was more cost-effective for 100% of the model iterations (including superior in 99% of iterations) against using the ZN microscopy (Fig 3). While compared to the use of Xpert, the MPT64 test was superior for 3% of iterations, and the output showed lower incremental cost and efficacy for an ICER above and below the WTP for 20% and 77% of the model iterations (Fig 4), respectively.

The one-way sensitivity analyses are presented in the tornado diagram (Fig P in S1 Text). The rise in ICER was most sensitive to the increase in the following variables: prevalence of HIV among EPTB cases, their probability of treatment, costs of ART as well as the probability of MPT64 test in detecting EPTB among HIV positive or negative patients. The variables whose decrease showed sensitivity to an increase in ICER were probabilities of Xpert positive among HIV negative or positive patients as well as probabilities of death among drug sensitive EPTB positive patients. None of the model range of variables’ values pushed the ICER above the assumed WTP threshold of USD 1790.

The successive stages of the Markov model simulations show the role of HIV and EPTB case classification in acquiring costs and QALYs. For the above-described baseline model set-up, over the lifespan using ZN microscopy, Xpert and MPT64 tests per patient, cost estimates were at USD 1556, 1210 and 1579 for the resulting QALYs of 11.04, 11.52 and 12.89, respectively. The cost and accuracy of HIV diagnosis and ART were modelled independent of using any EPTB diagnostic test. Due to the ART costs, the long-term cumulative cost per model set-up was higher for HIV-co-infected patients. Moreover, the non-TB/HIV positive patients had relatively higher long-term cumulative costs due to their lower mortality (only background mortality) compared to EPTB/HIV positive patients who have relatively higher disease-associated mortality (Figs Q-S in S1 Text). Extrapolating our results to a cohort of 10,000 would result in 6200, 6570 and 7530 patients receiving appropriate treatment following ZN microscopy, Xpert and MPT64 test strategies’ use, respectively. Using the Xpert test’s sensitivity of 0.49 and 0.79, our model output showed that its cost estimates changed to USD 1777 and 1947, resulting in 12.50 and 13.61 QALYs, respectively. In addition to the CEA per model output (Fig M in S1 Text) based on the above-described model input parameters (Table 1), the scenario analyses conducted to account for the higher diagnostic sensitivity of the Xpert test for lymph node aspirates, one of the common EPTB disease presentation [60]. Based on these values, the Markov model simulations showed that the Xpert test at a sensitivity probability of 0.49 and ZN microscopy were absolutely dominated by the MPT64 test (Fig T in S1 Text). While using the Xpert test’s sensitivity probability of 0.79 as the model input, it was the most cost-effective diagnostic test per model simulations (Fig U in S1 Text).

## Discussion

We find the MPT64 test a cost-effective diagnostic test compared to Xpert and ZN microscopy to diagnose previously untreated EPTB adult patients. This is the first study that evaluates the cost-effectiveness of an immunohistochemistry-based test such as MPT64 compared to Xpert and ZN microscopy used for EPTB diagnosis. Moreover, there are no earlier economic evaluations for EPTB patients using QALYs as an outcome. This study fills both these gaps. The model output generated an ICER, which is cost-saving. Therefore, at the baseline model inputs, the MPT64 test may be considered the most cost-effective test for the laboratory confirmation of presumptive EPTB cases, and sensitivity analyses confirm that this conclusion is robust. The use of QALYs as an outcome will also help to compare the results of this study with other economic evaluations and encourage broader consideration of EPTB diagnostics as part of healthcare priority settings.

We accounted for uncertainty in the model parameters and presented cost-effectiveness against the assumed WTP. The finding that the Xpert test is not the most cost-effective compared to other diagnostic tests differs from the published economic evaluations of TB diagnostics. However, such literature is focused on pulmonary TB, not EPTB [12, 13, 28–30, 33]. Unfortunately, the economic evaluations for diagnostic tests for EPTB are not available in published literature. This prevents us from comparing our study with the existing literature primarily focused on pulmonary TB. However, while using the Xpert test’s higher probability of diagnostic test sensitivity, the test appeared to be the most cost-effective diagnostic test.

This use of a single CRS to validate all three diagnostic tests in our study stands out in the existing economic evaluation literature. Instead of relying solely on crude assumptions for disease prevalence and diagnostic tests’ accuracies, we modelled the empirically validated diagnostic properties of Xpert, ZN microscopy and MPT64 tests.[7, 15] Adopting newer diagnostics would also address the conditional recommendation amid very low-quality evidence for Xpert use for several forms of EPTB illness [1, 12–14]. However, it is important to note that the MPT64 test also needs a minimal set-up, including a collection of biological specimens and processing common to all diagnostic test strategies.

It is essential to highlight the key assumptions or limitations of this model. The study focused on previously untreated EPTB adult patients and did not account for relapse of illness or retreatment. EPTB patients having a relapse of illness would have different disease pathogenesis, health utility and costs that are not covered in this analysis. The diagnostic tests’ accuracy parameter and mortality rates used in our model reflect the most common forms of EPTB illness, mainly lymphadenitis as well as pleural and abdominal sites of infection, rather than the less prevalent tuberculosis meningitis. However, for scenario analyses, we have also used higher Xpert test sensitivity rates based on different sites of infections reported in a literature review [60]. Based on our cohort studies, we used the CRS as a reference standard to estimate the diagnostic tests’ accuracies. The results from scenario analyses should be used carefully, as the parent studies did not provide detailed information about the CRS used to estimate diagnostic test accuracies. Due to the varying disease spectrum and paucibacillary nature of EPTB disease, the affected patients classified by a CRS or bacterial culture results as reference standards may have a low yet existing probability of incorrect classification. However, the CRS used in this economic evaluation includes clinical signs and symptoms, response to treatment, culture, and other criteria such as bacteriological, radiological, radiological, cytological, and histological confirmation, published elsewhere [7]. Due to the non-availability of a uniform CRS for EPTB case classification, it is not possible to have a standardised CRS to assess the diagnostic accuracy of laboratory tests under evaluation.

Our model assumed a similar compliance of ATT for all three test strategies. Using the MPT64 test to diagnose EPTB is a relatively newer approach with diagnostic input parameters based on our prospective cohort study (Table 1). The cost of collecting and processing biological specimens before laboratory confirmation by the diagnostic tests under evaluation in our study would not affect our measure of interest, the ICER. Therefore, in this modelling evaluation, we did not include these costs. An implication of this approach may be the limited generalizability of our results in healthcare settings that markedly vary from Tanzanian settings. EPTB illness is a constellation of signs and symptoms that vary with the affected body site. Therefore, when unavailable, our model parameters had to be assumed in discussion with the clinical experts (Table 1). It would be unrealistic to parametrise the model based on other diseases, including pulmonary TB, as it varies widely from EPTB. There is no reported data about the long-term values of health utilities of EPTB patients (regardless of HIV status). Therefore, the value of health utilities among EPTB negative asymptomatic HIV patients on ART may have some limitations. However, a meta-analysis reported similar high health utilities (0.94) for such HIV patients since the advent of modern ARTs that have markedly improved up till now [56]. Per available literature and disease pathogenesis, EPTB is not associated with very high mortality; however, we assumed a slightly higher probability of mortality in their respective categories for patients (either DST/MDR having/not having HIV) if they did not receive treatment (Table 1).

Our analysis models patients who had not received EPTB treatment during the previous year. Most of these patients may be the newly affected ones. Therefore, the study may not be generalisable to a more complicated progression of EPTB illness, usually among relapse of an infection. This economic evaluation considered costs from a healthcare perspective. However, TB patients may also bear indirect costs in accessing healthcare, especially in the low-middle income setting, which should also be studied in future studies from a patient’s perspective. To date, the prevalence of MDR has not been reported as a significant challenge among previously untreated EPTB patients, and there is limited evidence of MDR among such patients. However, we retrieved the probability of MDR among such EPTB patients from a cohort study in Pakistan and used it in this model [4, 42]. The Markov model pathways of MDR patients are not reported; thus, we adapted the pathways of drug-sensitive patients. It is important to note that, unlike Xpert, both ZN microscopy and MPT64 tests do not have the inherent ability to test drug sensitivity. Therefore, the benefits of getting appropriately tested and treated for MDR were only included for the Xpert test usage.

Finally, this study shows that healthcare providers can offer EPTB patients a cost-effective option with sensitive diagnostics. For example, the MPT64 test identified EPTB cases with the highest probability of true positive and lowest of false negative test results. We suggest exploring the adoption of newer tests such as MPT64 and strengthening EPTB diagnostic laboratory services. Moreover, Xpert appearing as the most cost-effective option during a PSA scenario (with a higher sensitivity) may reflect a potential use of various diagnostic tests based on different sites of infection and should be considered as part of EPTB diagnostic pathways. This will provide an opportunity for cost-effective diagnosis as a first step of EPTB treatment for those in need, and hence, fits well with the WHO 2020 End TB Strategy. Moreover, not detecting EPTB would also add to disease prevalence and accompanying impacts, such as lower health utility.

## Supporting information

S1 Text (DOCX)

S1 Dataset (XLSX)

S2 Dataset (SAV)
