# Supplementary material for: Comparing the cost-effectiveness of the MPT64-antigen detection test to Xpert MTB/RIF and ZN-microscopy for the diagnosis of Extrapulmonary Tuberculosis: An economic evaluation modelling study
Source: PLOS Glob Public Health. 2024 Aug 8;4(8):e0003414. doi: 10.1371/journal.pgph.0003414 (PMC11309377; doi:10.1371/journal.pgph.0003414)
Supplement: S1 Text — (DOCX) [file pgph.0003414.s001.docx]

**Supplementary file**

**Health-related Quality of Life data**

The patient-level EuroQol 5 dimensions 3-levels (EQ-5D-3L) data used in this study to estimate health state and single utility have already been published as part of our findings from the cohort study in Zanzibar, Tanzania.(1) For our economic evaluation model, we used the health utility data by HIV subgroups (see the manuscript and Table S1).

**Table A: Health profiles as reported by the EPTB patients by HIV subgroups based on EQ-5D-3L before and after treatment in Zanzibar.**

|  | | **Health states** | **Frequency** | **Percent** | **Cumulative**  **percent** | **Health utilities** |
| --- | --- | --- | --- | --- | --- | --- |
| **HIV negative**  **(n= 26)** | **Before treatment** | 11111 | 6 | 23.08 | 23.08 | 1 |
|  |  | 11121 | 2 | 7.69 | 30.77 | 0.83 |
|  |  | 11122 | 1 | 3.85 | 34.62 | 0.79 |
|  |  | 11212 | 1 | 3.85 | 38.46 | 0.81 |
|  |  | 11221 | 2 | 7.69 | 46.15 | 0.79 |
|  |  | 11222 | 1 | 3.85 | 50 | 0.74 |
|  |  | 12122 | 1 | 3.85 | 53.85 | 0.69 |
|  |  | 12211 | 1 | 3.85 | 57.69 | 0.76 |
|  |  | 12311 | 1 | 3.85 | 61.54 | 0.67 |
|  |  | 21222 | 1 | 3.85 | 65.38 | 0.69 |
|  |  | 21321 | 1 | 3.85 | 69.23 | 0.64 |
|  |  | 21322 | 1 | 3.85 | 73.08 | 0.60 |
|  |  | 22212 | 1 | 3.85 | 76.92 | 0.66 |
|  |  | 22221 | 1 | 3.85 | 80.77 | 0.64 |
|  |  | 22222 | 2 | 7.69 | 88.46 | 0.60 |
|  |  | 32322 | 1 | 3.85 | 92.31 | 0.36 |
|  |  | 33322 | 2 | 7.69 | 100 | 0.22 |
|  | **After treatment** | 11111 | 22 | 84.62 | 84.62 | 1 |
|  |  | 11121 | 1 | 3.85 | 88.46 | 0.83 |
|  |  | 12111 | 1 | 3.85 | 92.31 | 0.81 |
|  |  | 21111 | 1 | 3.85 | 96.15 | 0.84 |
|  |  | 22221 | 1 | 3.85 | 100 | 0.64 |
| **HIV positive**  **(n= 5)** | **Before treatment** | 11221 | 1 | 20 | 20 | 0.79 |
|  |  | 11321 | 1 | 20 | 40 | 0.70 |
|  |  | 21322 | 1 | 20 | 60 | 0.60 |
|  |  | 22122 | 1 | 20 | 80 | 0.64 |
|  |  | 33322 | 1 | 20 | 100 | 0.22 |
|  | **After treatment** | 11111 | 5 | 100 | 100 | 1 |

The five dimensions of the EQ-5D-3L include Mobility, Self-care, Usual Activities, Pain/Discomfort, and Anxiety/Depression, which have three levels of response: from no problems to extreme problems (labelled as 1-3). These levels of each dimension can be combined to identify 243 possible health states from 11111 (full health) to 33333 (worst health).(2) By using these scores for EQ-5D-3L profiles, each health state can be used to define one single “utility” score. In the current study, due to the unavailability of the Tanzanian value set, we used the Zimbabwe value sets.(3) These value sets have been validated, and corresponding methods to estimate health utility as proposed by the EuroQol are published elsewhere.(3).(4) For example, we subtracted the value of 1 corresponding to full health by 0.10 if at least one 2 or 3 was among the health profile. Following that we subtracted the remainder according to the Zimbabwean TTO value set corresponding to the health profile reported by the individual patient (table S2).(4)

As a standard method of utility estimation per EQ-5D-3L health profiles, we deducted the value of the full health state (equal to 1) for the corresponding value of an individual’s health profile of the five dimensions. Then we used mean values of the estimated utilities before and after the treatment by HIV subgroups in our economic evaluation model.

**Table B: Zimbabwean value set to estimate health utility from patient-reported health profiles.**

|  | Health profile | Decrement from full health (1) |
| --- | --- | --- |
|  | At least one 2 or 3 (constant)  3 (N3) | - 0.100 |
| Mobility | 2 | - 0.056 |
|  | 3 | - 0.204 |
| Self-care | 2 | - 0.092 |
|  | 3 | - 0.231 |
| Usual activities | 2 | - 0.043 |
|  | 3 | - 0.135 |
| Pain/discomfort | 2 | - 0.067 |
|  | 3 | - 0.302 |
| Anxiety/depression | 2 | - 0.046 |
|  | 3 | - 0.173 |

**Adjustment of cost parameter considering the inflation rate**

To standardize the cost inputs to a single base year, we converted all costs equivalent to USD 2021 using USD 122 as the latest available the World Bank’s annual GDP deflator for Tanzania.(5) To come up with the cost parameter as *X* for 2021 as the single base year, we adopted the standard methodology.(6)

$$X=\left( A \left( cost in USD \right)* \frac{r}{z} \right)$$

$$GDP deflator base year (r)=122$$

$$GDP deflator previous year=z$$

$$Previously known cost of item=A$$

$$Adjusted cost value of item=X$$

**Table C: Cost parameters adjusted using the latest available (year 2021) the World Bank’s annual GDP deflator for Tanzania.**

| **Utilities** | **Costs (literature-based)**  ***A*** | **Previous year when costing parameter was estimated** | **GDP deflator for the previous year**  ***z*** | **Base year (2021) cost estimate**  ***X*** |
| --- | --- | --- | --- | --- |
| Cost of Xpert test | 19 | 2012 | 80 | 29 |
| Cost of HIV diagnostics | 31 | 2018 | 114 | 33 |
| Cost of ZN Microscopy | 13 | 2018 | 114 | 14 |
| Cost of first-line TB treatment | 135 | 2018 | 114 | 144 |
| Cost of second-line TB treatment | 3230 | 2018 | 114 | 3457 |
| Cost of HIV treatment | 265 | 2012 | 80 | 404 |
| Cost of HIV treatment in loss-to-follow up patients | 107 | 2012 | 80 | 163 |


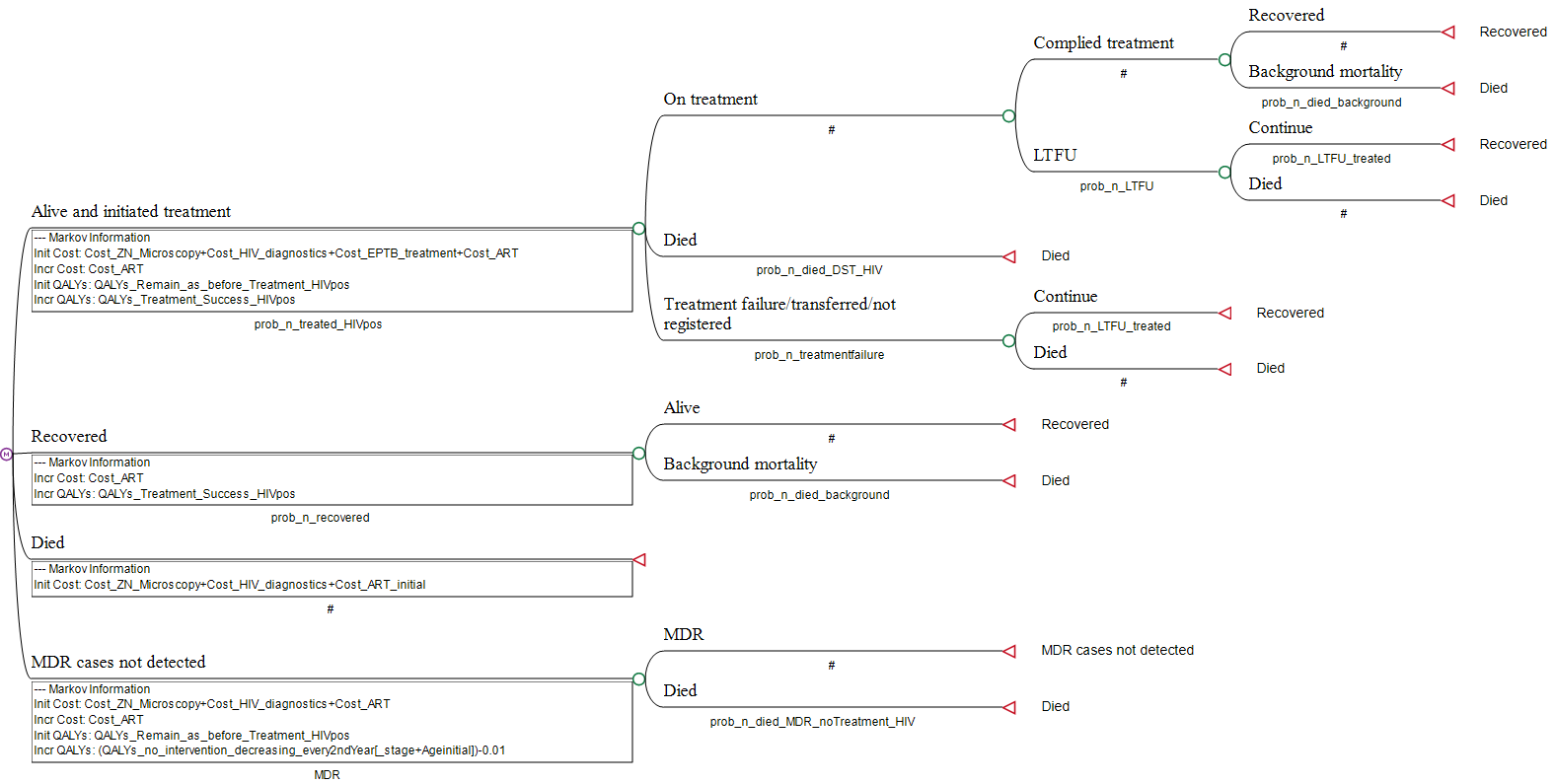


**Fig A. The Markov model illustrates long-term outcomes and flow of EPTB patients in the true positive (TP) and HIV positive group using ZN microcopy or MPT64 tests.**


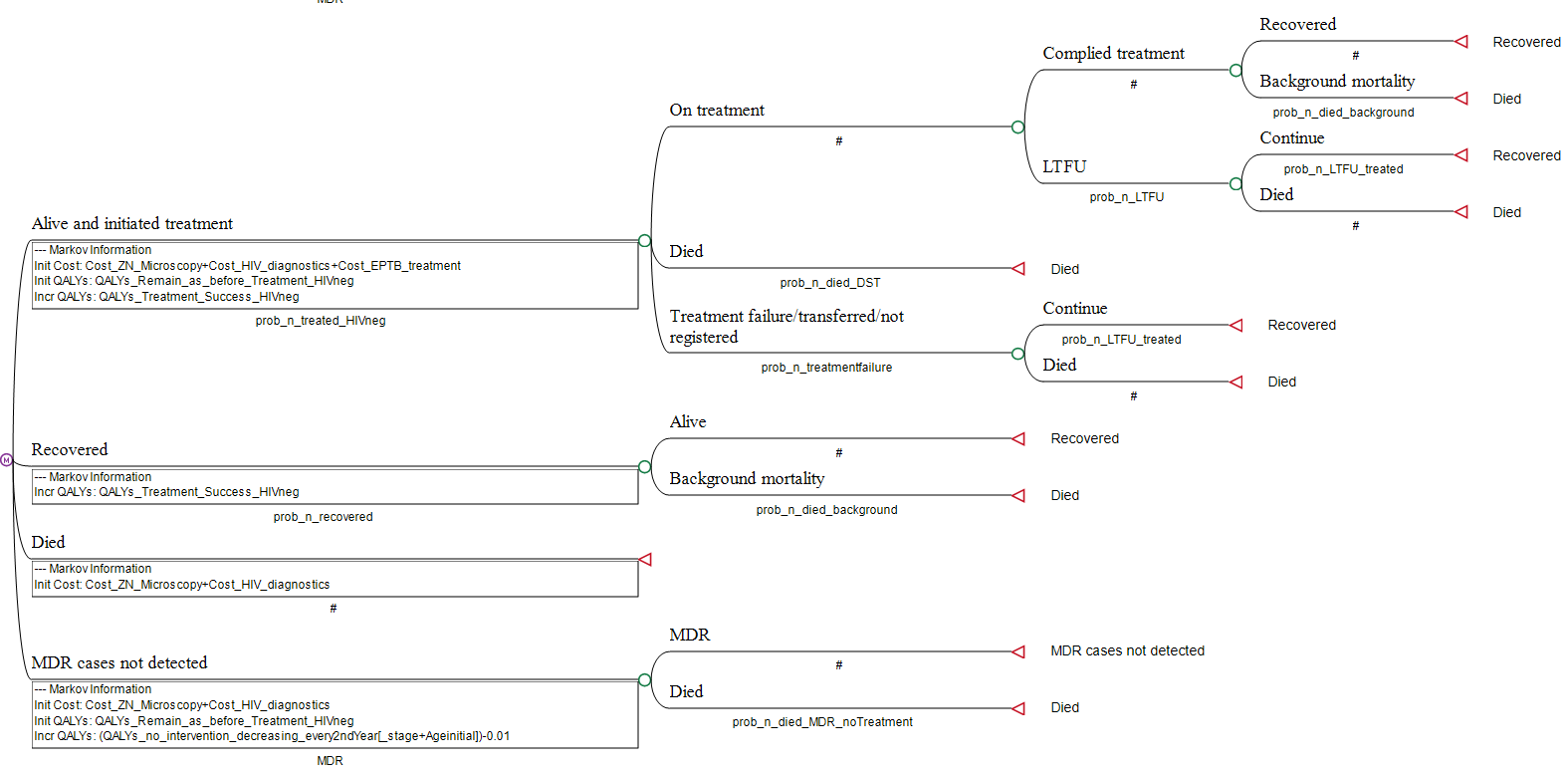


**Fig B. The Markov model illustrates long-term outcomes and flow of EPTB patients in the true positive (TP) and HIV negative group using ZN microcopy or MPT64 tests.**


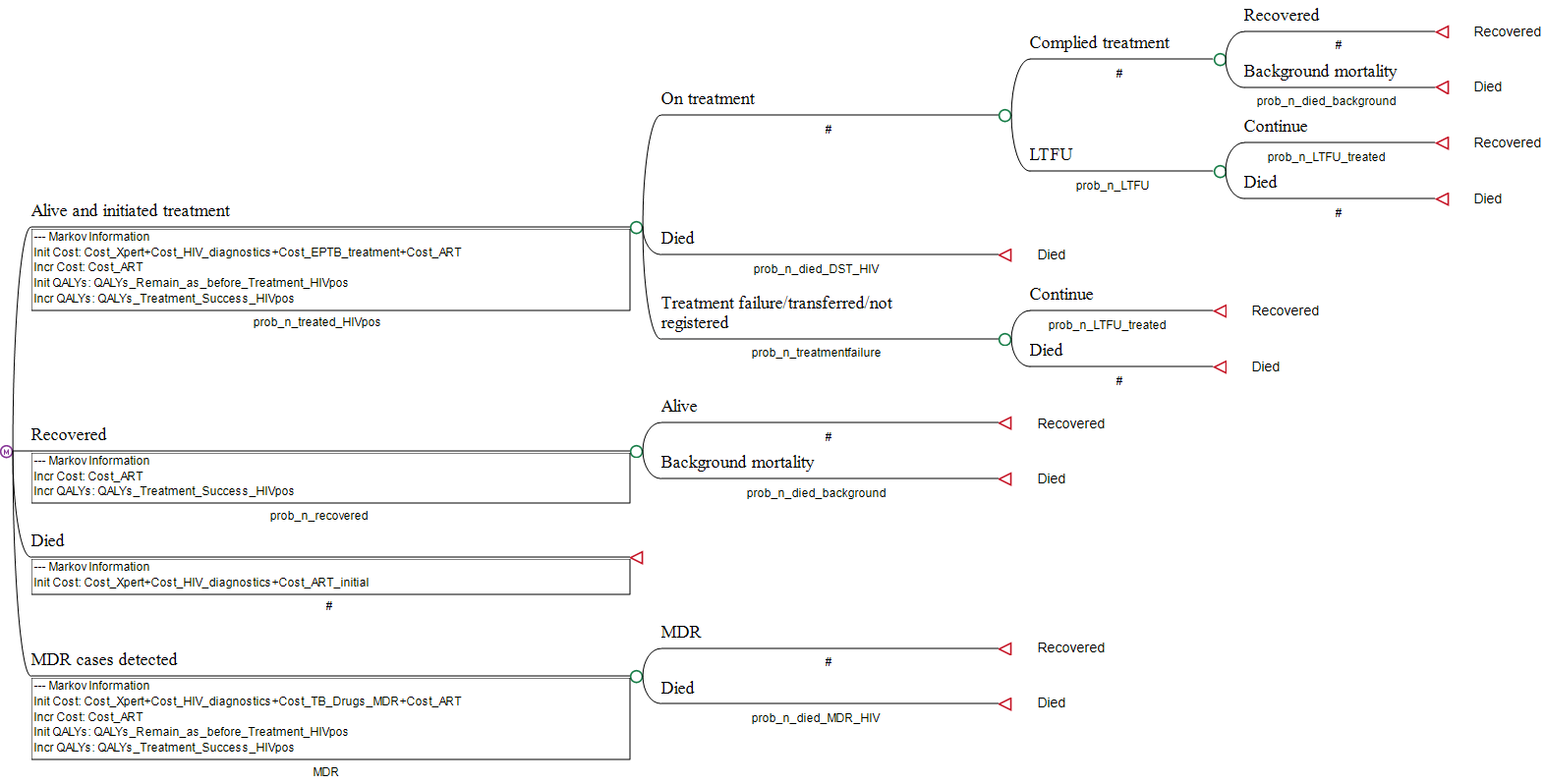
**Fig C. The Markov model illustrates long-term outcomes and flow of EPTB patients in the true positive (TP) and HIV positive group using Xpert test.**


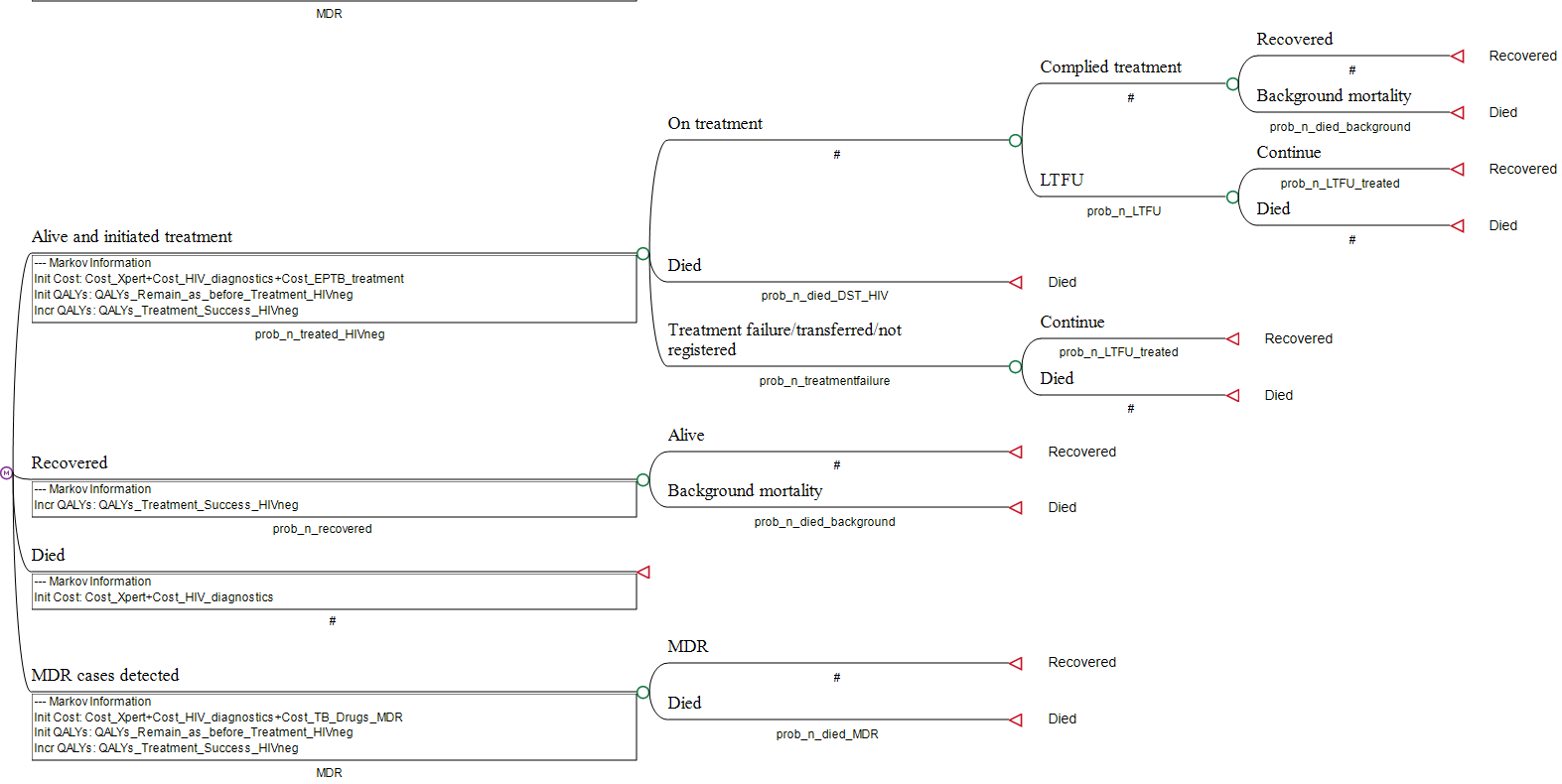
**Fig D. The Markov model illustrates long-term outcomes and flow of EPTB patients in the true positive (TP) and HIV negative group using Xpert test.**


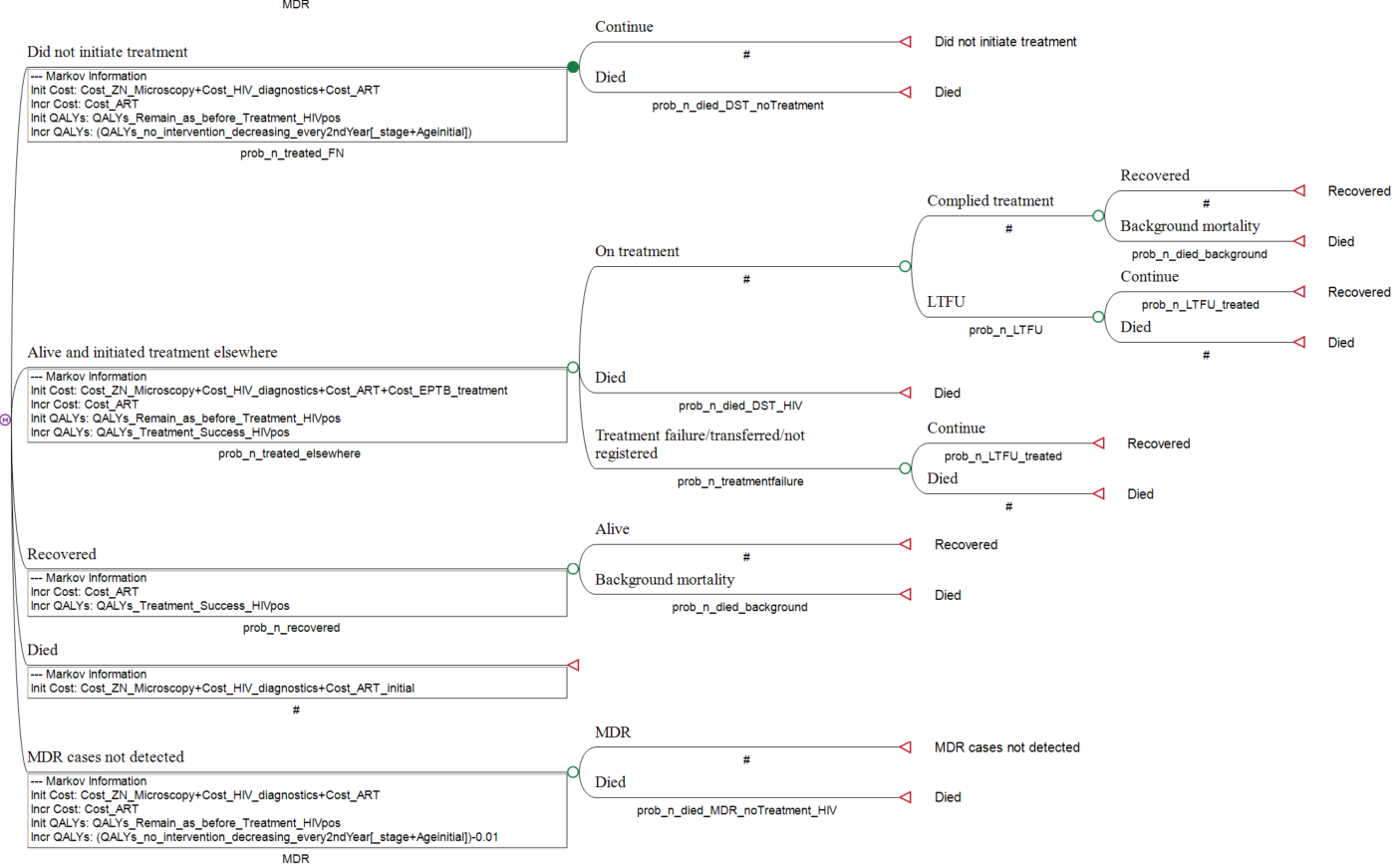


**Fig E. The Markov model illustrates long-term outcomes and flow of EPTB patients in the false negative (FN) HIV positive group using ZN microscopy or MPT64 test.**


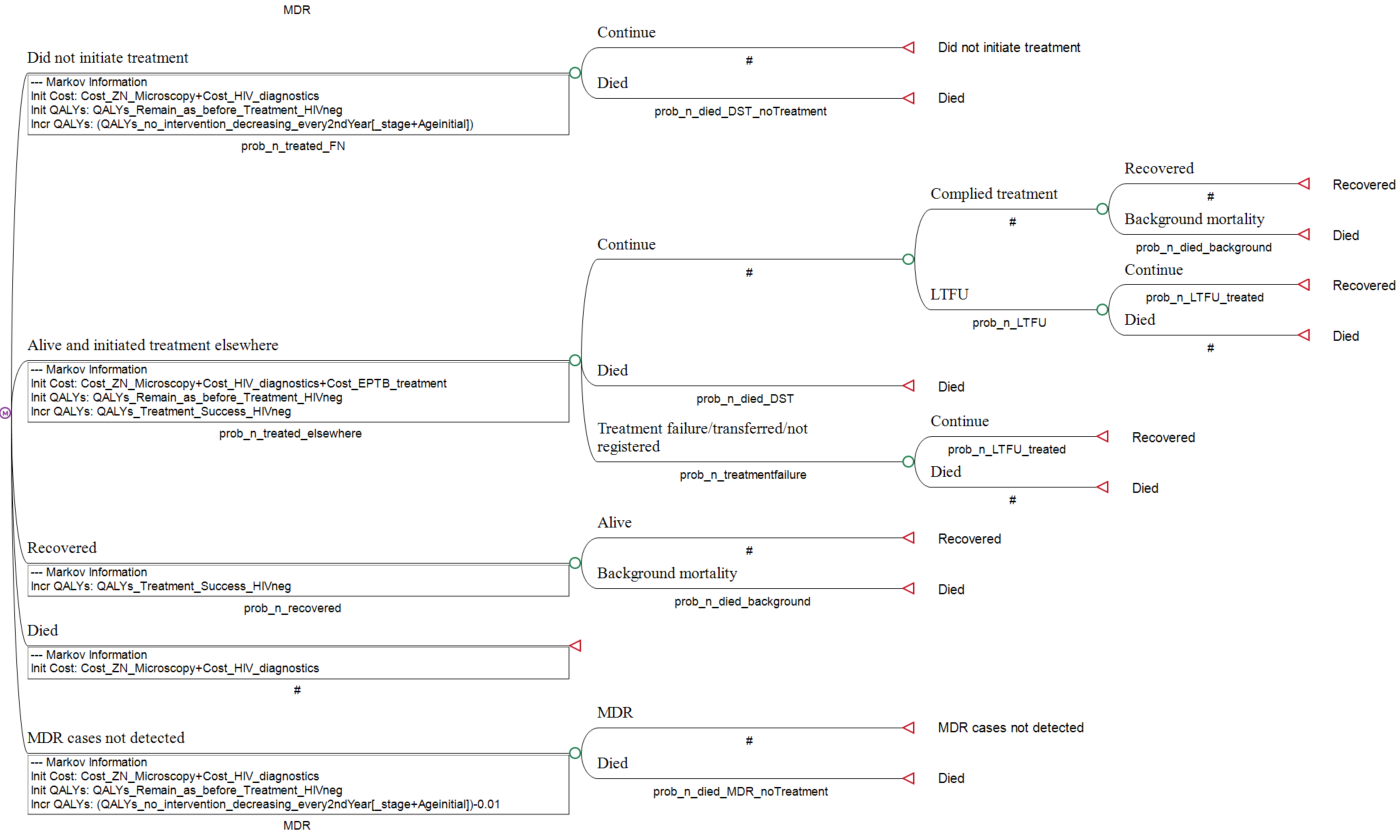
**Fig F. The Markov model illustrates long-term outcomes and flow of EPTB patients in the false negative (FN) HIV negative group using ZN microscopy or MPT64 test.**


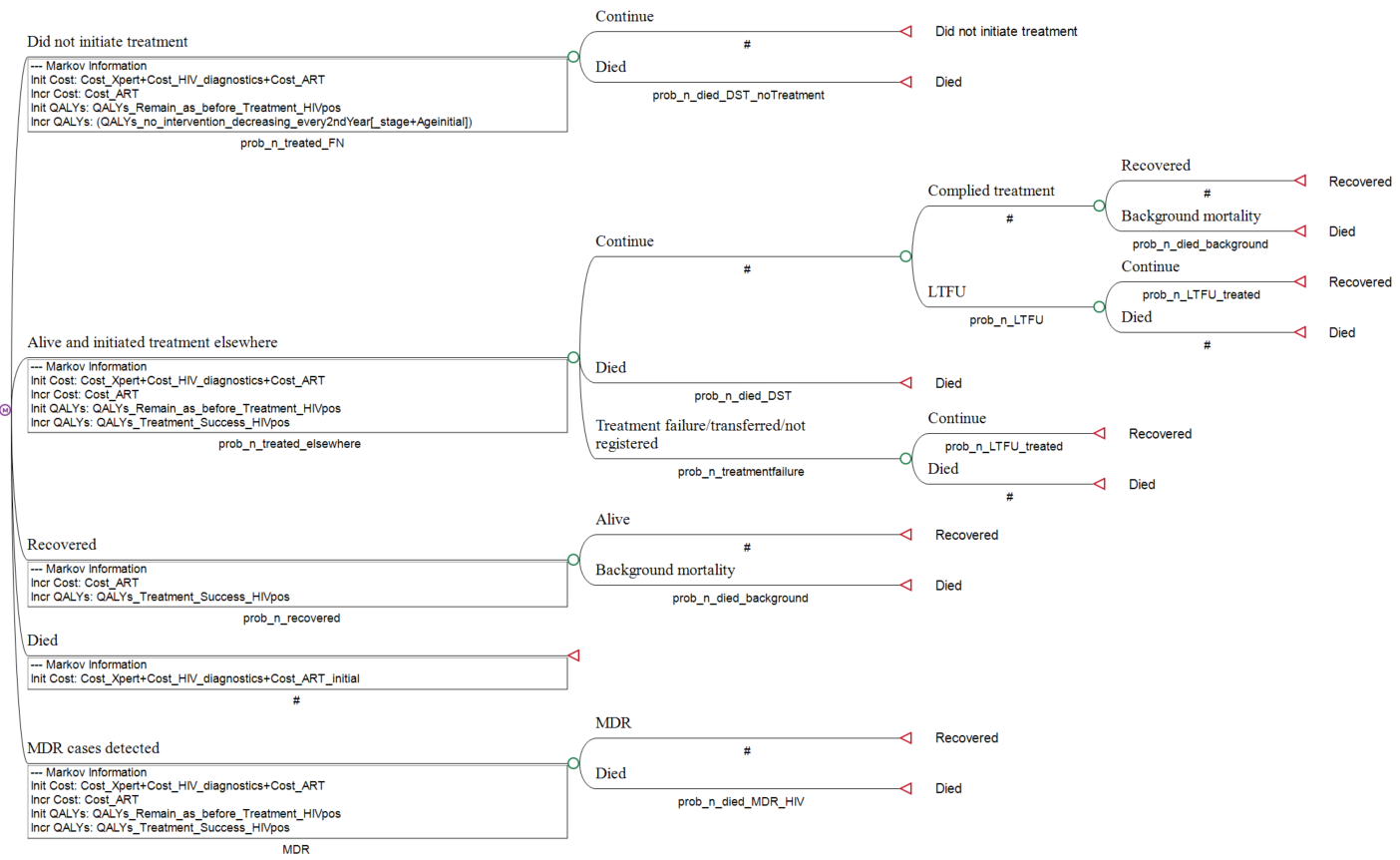
**Fig G. The Markov model illustrates long-term outcomes and flow of EPTB patients in the false negative (FN) HIV positive group using Xpert.**


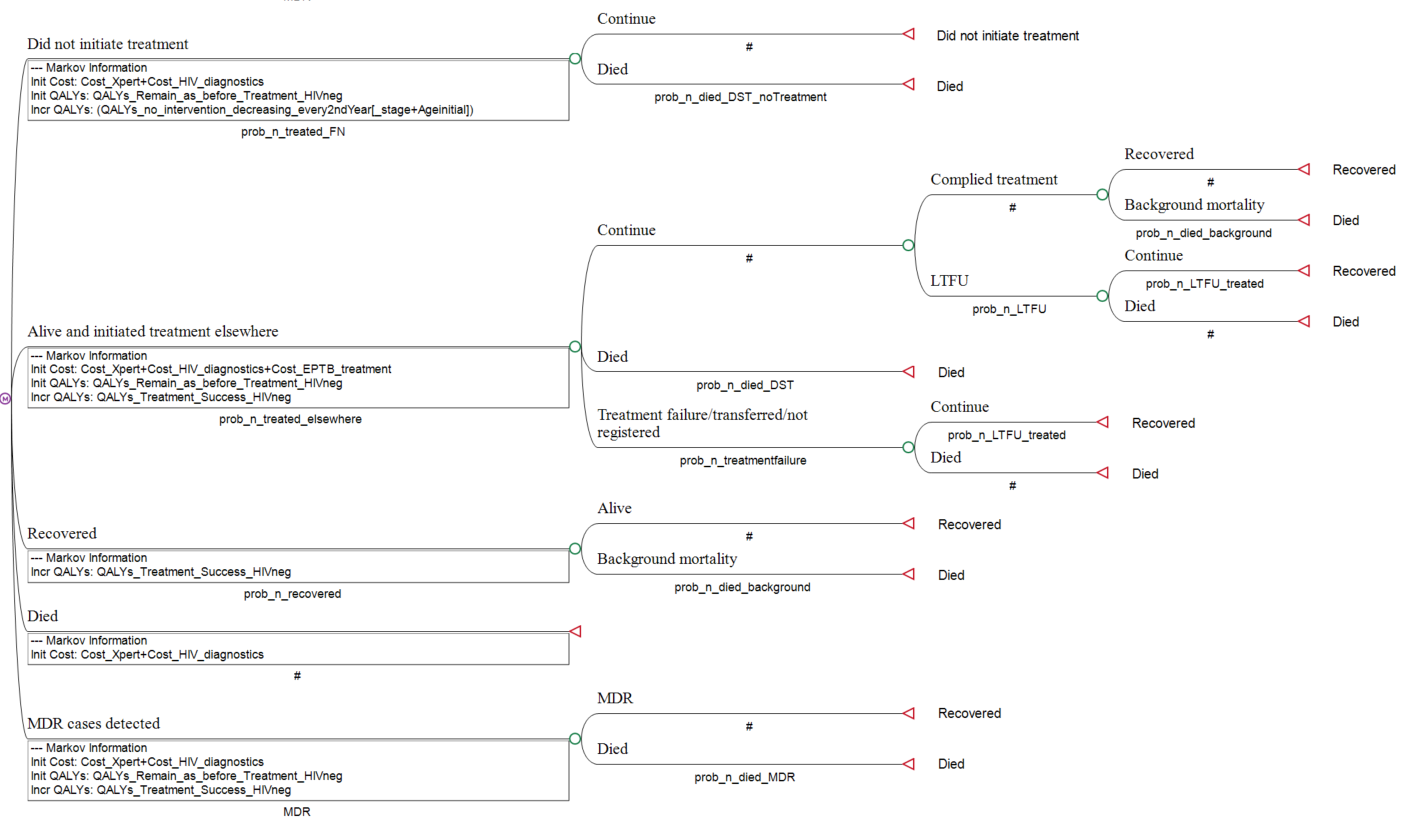


**Fig H. The Markov model illustrates long-term outcomes and flow of EPTB patients in the false negative (FN) HIV negative group using Xpert.**


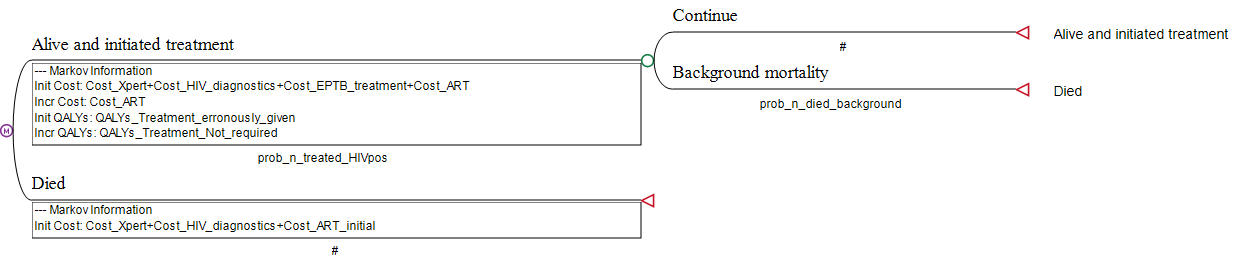
**Fig I. The Markov model illustrates long-term outcomes and flow of EPTB patients in the false positive (FP) and HIV positive group using either of the intervention.**


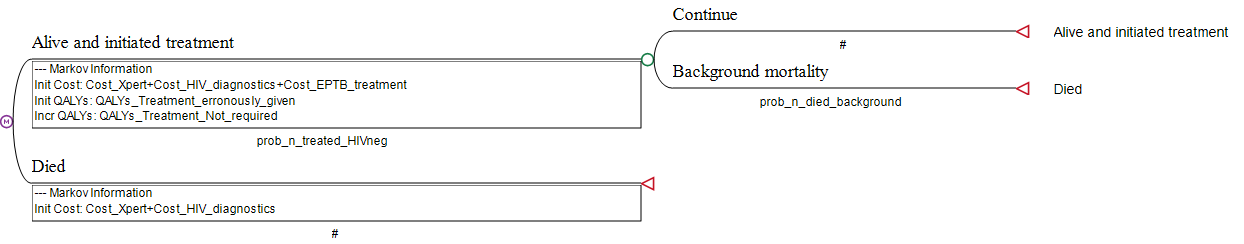


**Fig J. The Markov model illustrates long-term outcomes and flow of EPTB patients in the false positive (FP) and HIV negative group using either of the intervention.**


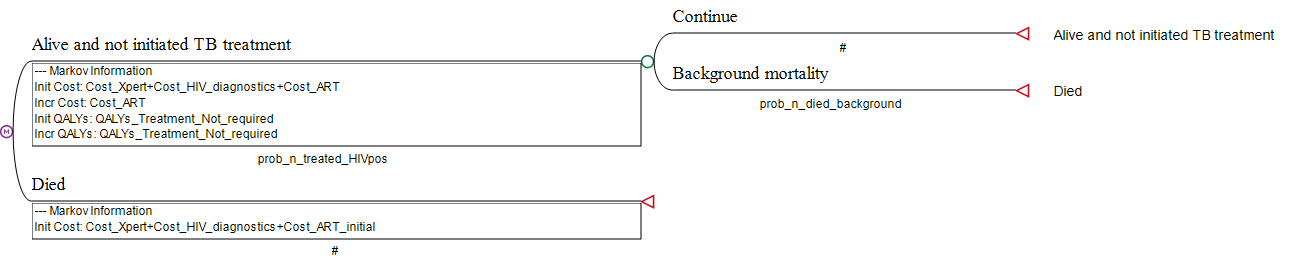
**Fig K. The Markov model illustrates long-term outcomes and flow of EPTB patients in the true negative (TN) and HIV positive group using either of the intervention.**


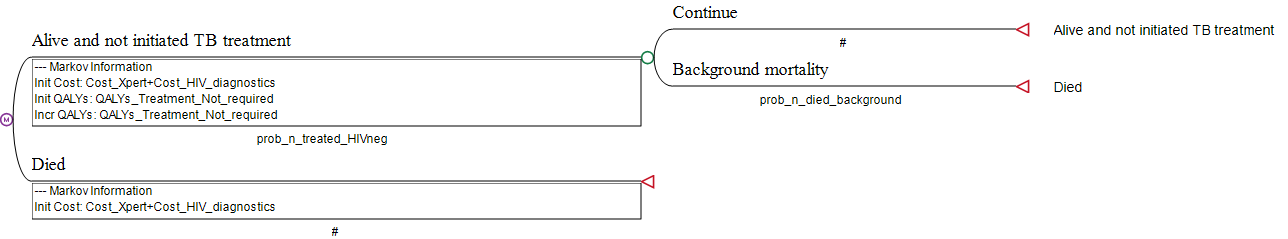
**Fig L. The Markov model illustrates long-term outcomes and flow of EPTB patients in the true negative (TN) and HIV negative group using either of the intervention.**


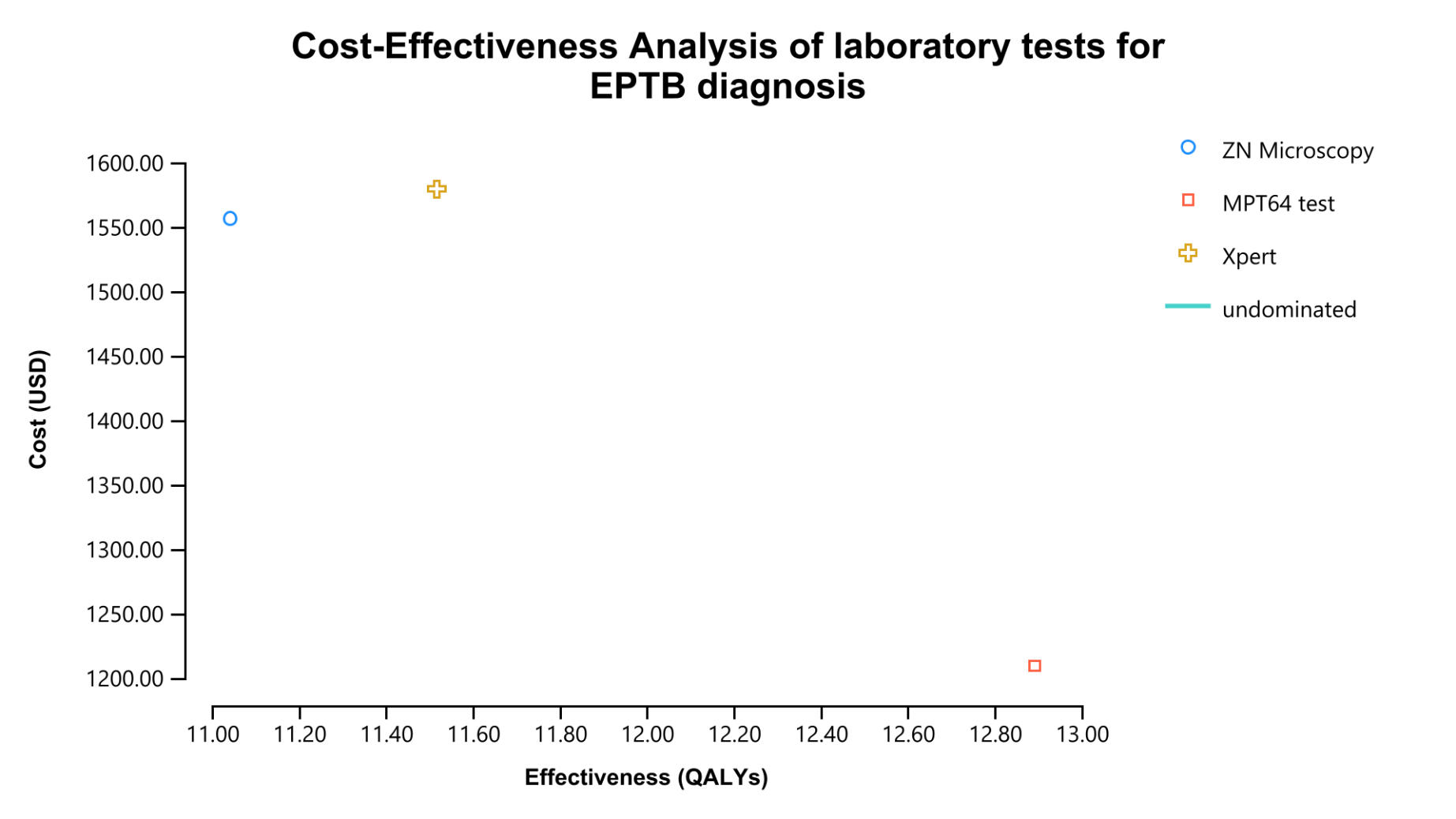


**Fig M. Cost-effectiveness plane showing the Markov model PSA per model input at baseline (tables 1-3).**


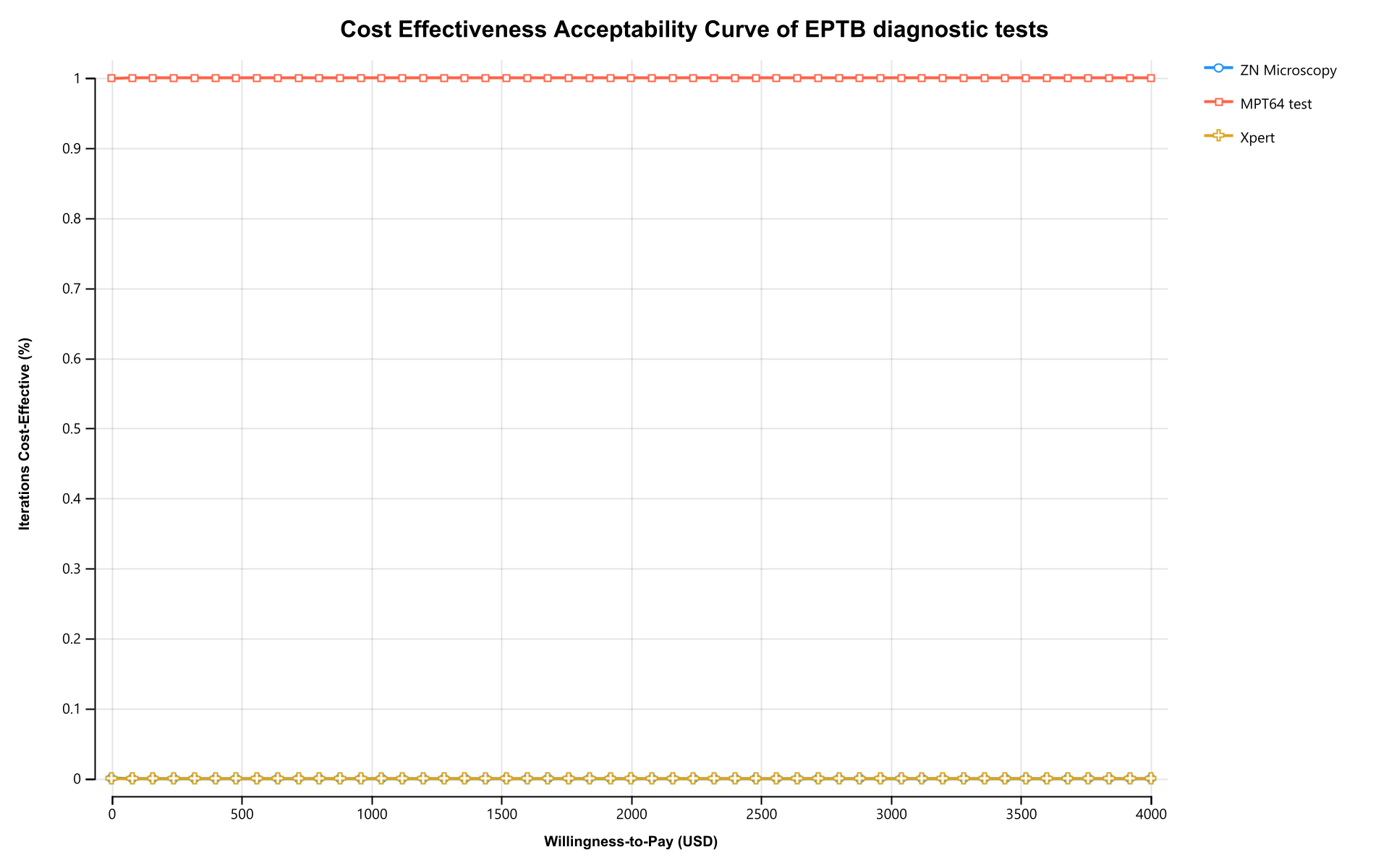


**Fig N. Cost-Effectiveness Acceptability Curve presenting the baseline model iterations depicting cost-effective diagnostic test against a range of willingness to pay thresholds (WTP).**


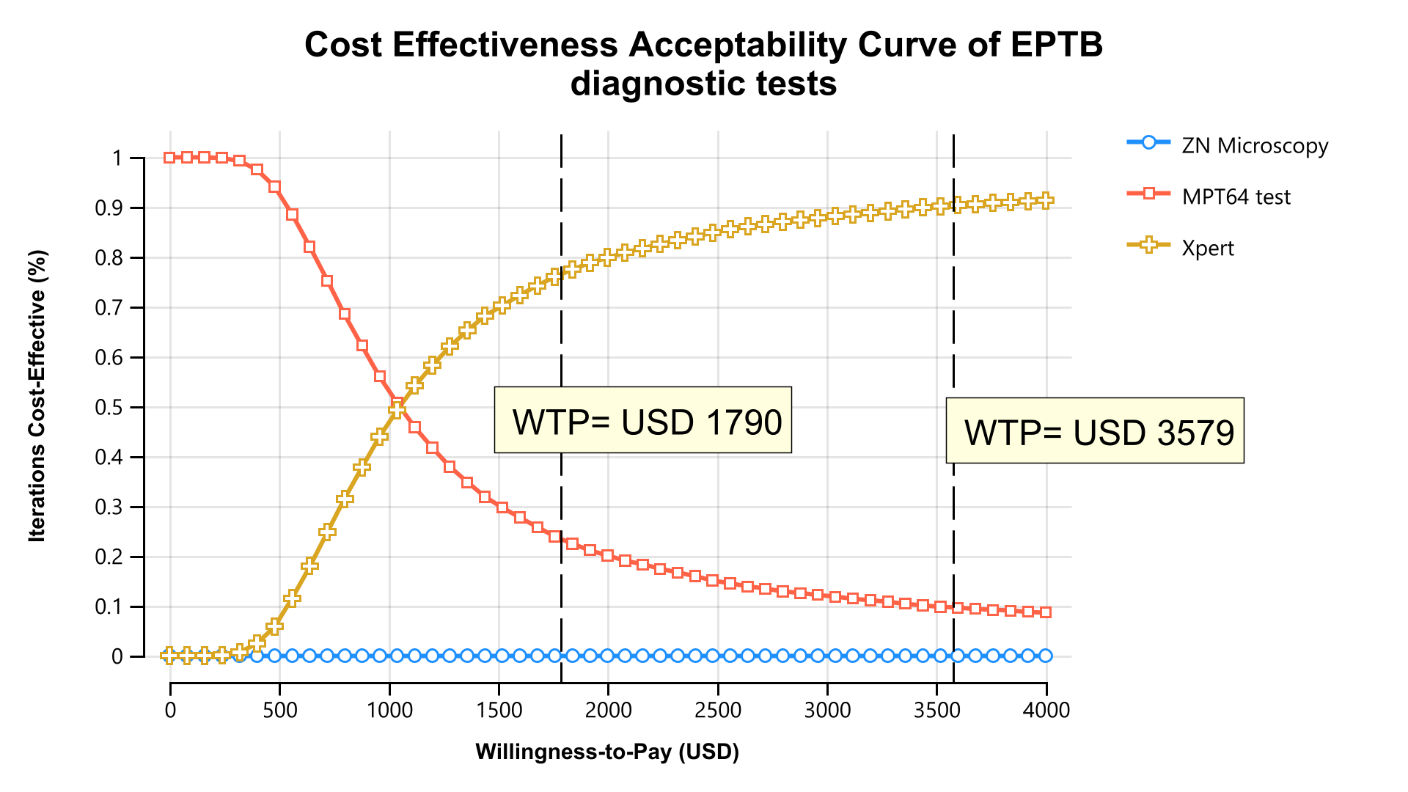


**Fig O. Cost-Effectiveness Acceptability Curve presenting the model iterations based on the higher Xpert test sensitivity (0.79%) depicting cost-effective diagnostic test against a range of willingness to pay thresholds (WTP).**


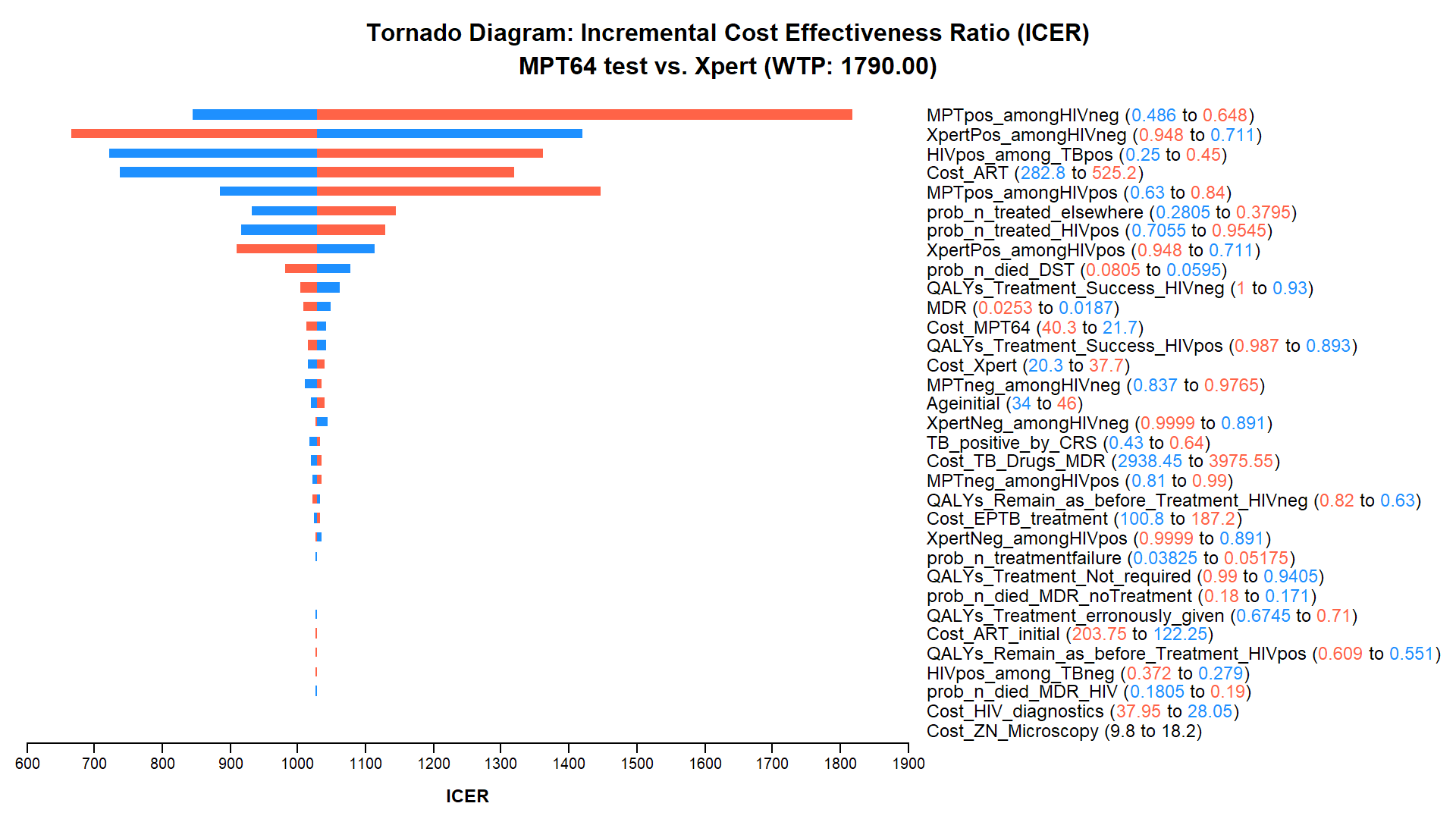


**Fig P. One-way sensitivity analyses of incremental cost-effectiveness, comparing MPT64 antigen detection test with the Xpert.**

Bars represent the ICER variation in relation to the high and low value of each parameter shown. Blue and red parts of the bar represent lower and higher values, respectively (for the rationale of for parameters’ range visit the methods section).


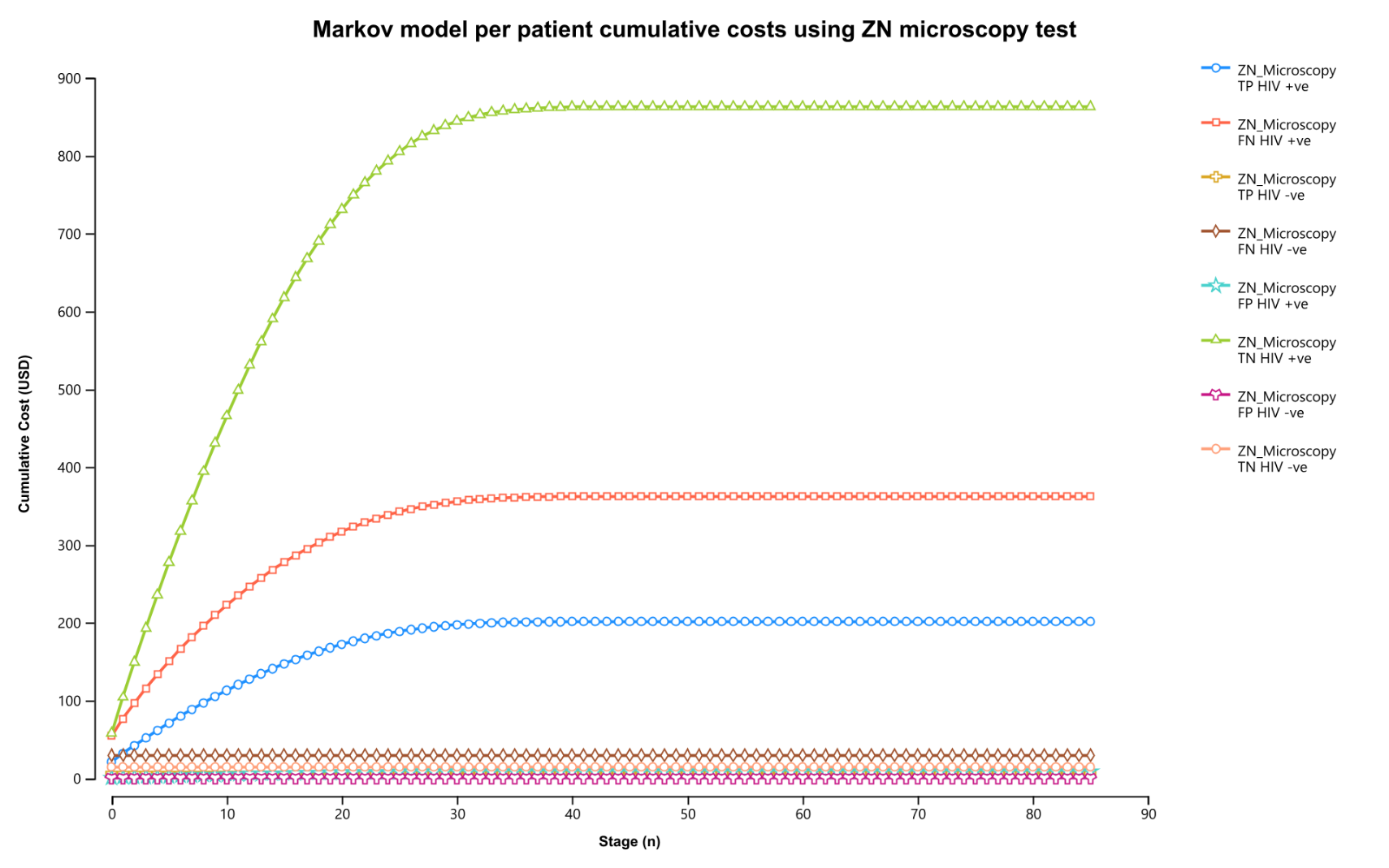


**Fig Q. Per patient long term cumulative costs using ZN microscopy test as diagnostic test for EPTB-HIV co-infected patients.**


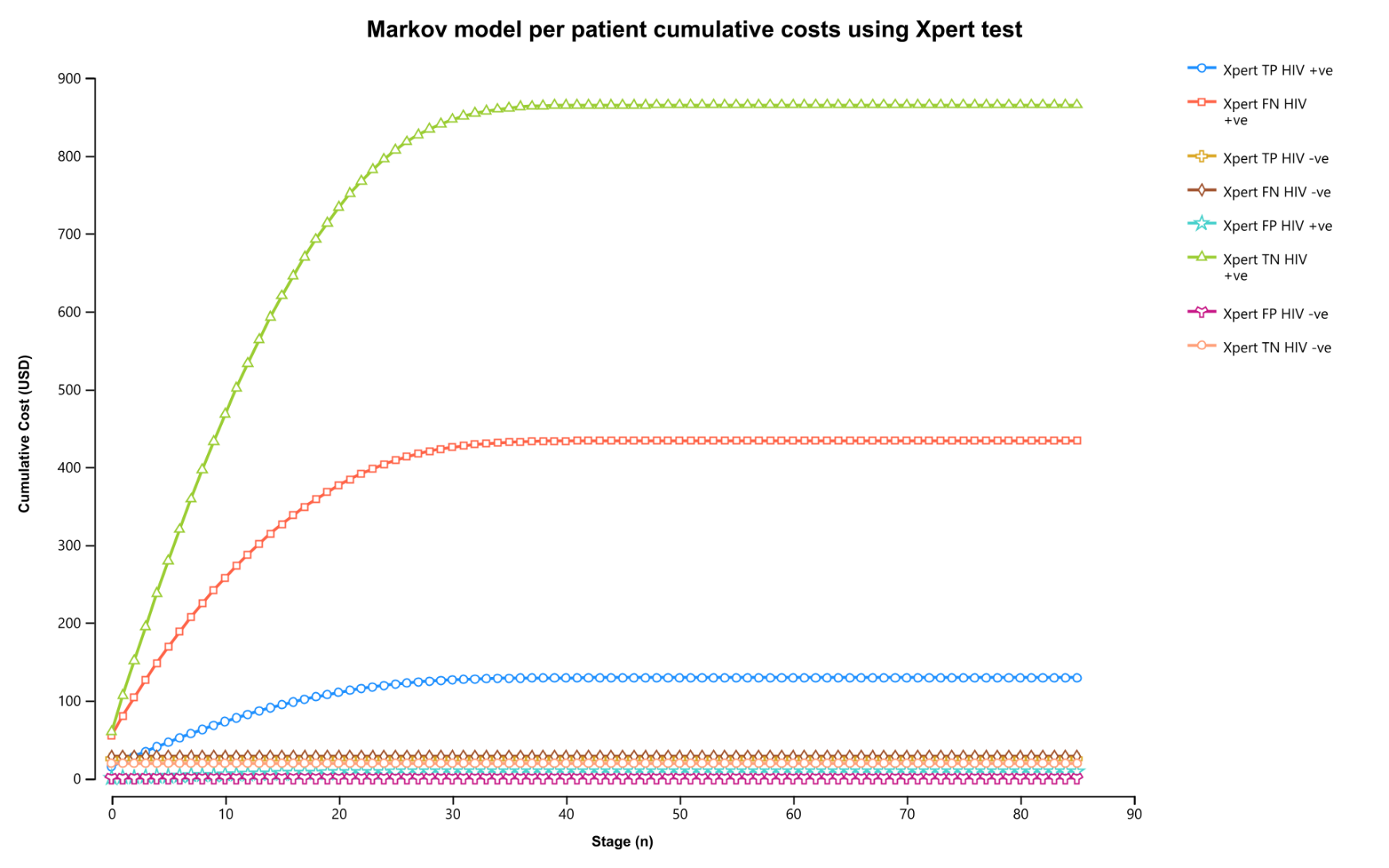


**Fig R. Per patient long term cumulative costs using Xpert test as diagnostic test for EPTB-HIV co-infected patients.**


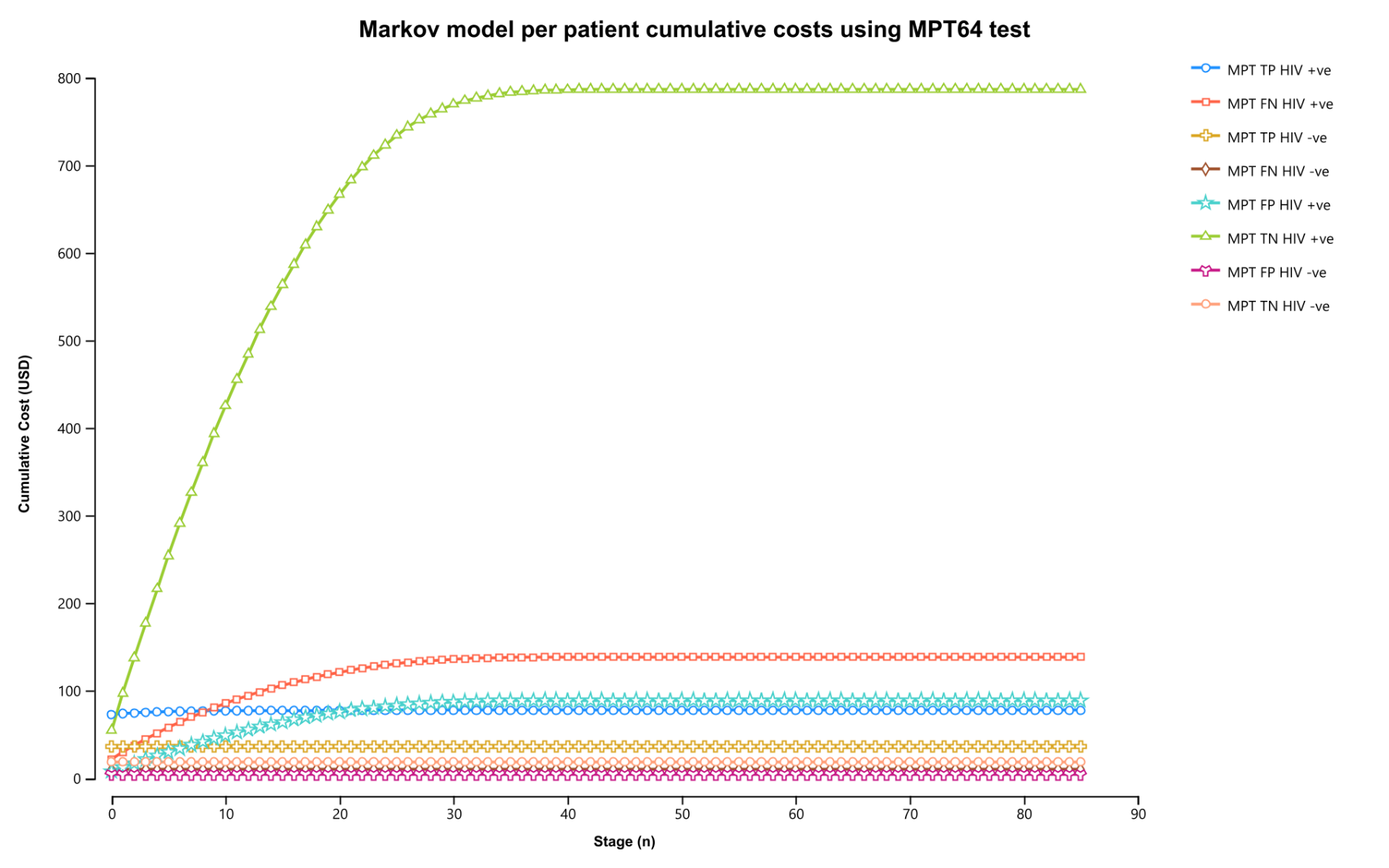


**Fig S. Per patient long term cumulative costs using MPT64 test as diagnostic test for EPTB-HIV co-infected patients.**


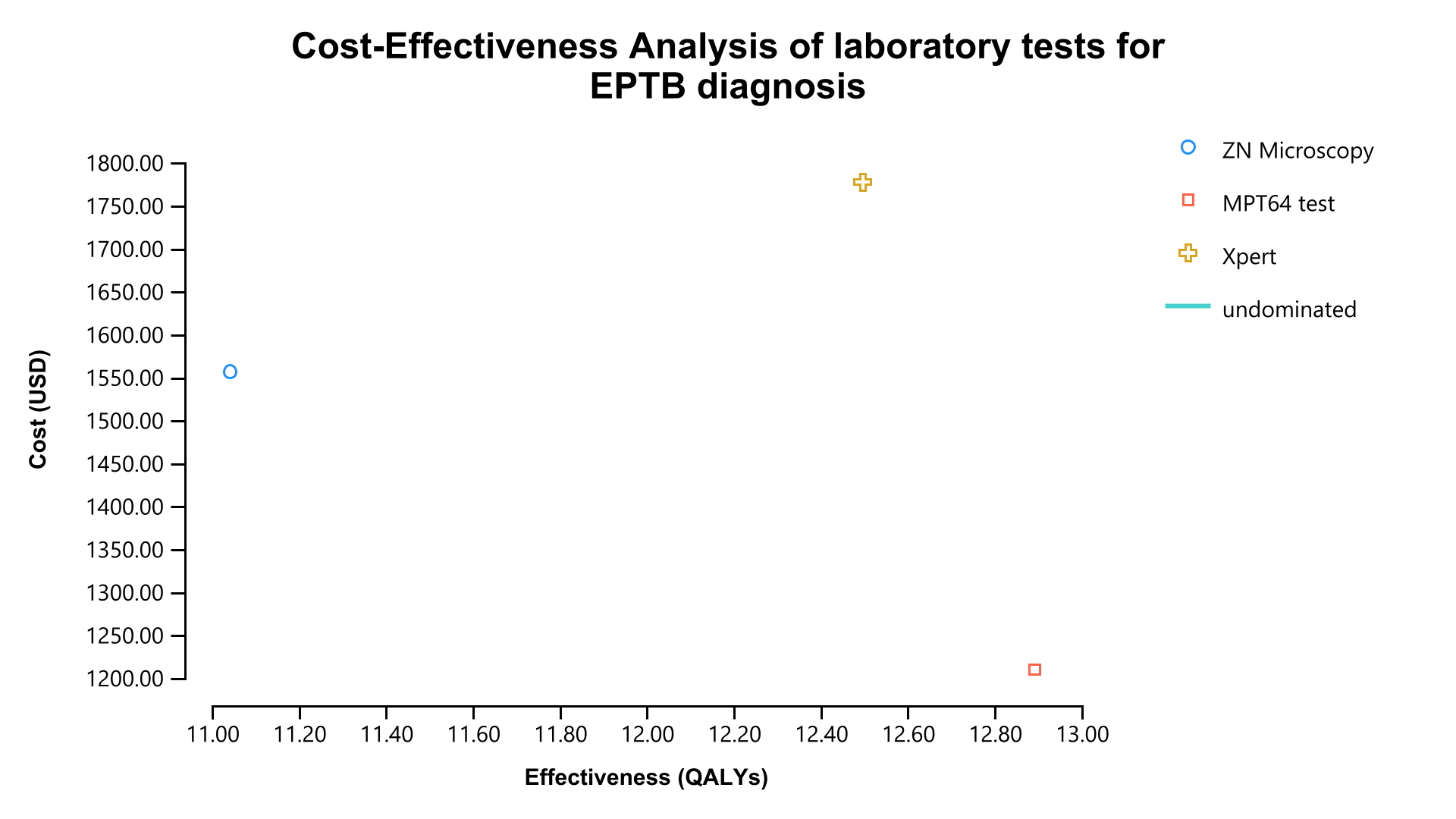


**Fig T. Cost-effectiveness plane showing the Markov model PSA per model input at baseline (tables 1-3), except probability of Xpert test sensitivity at 0.49.**


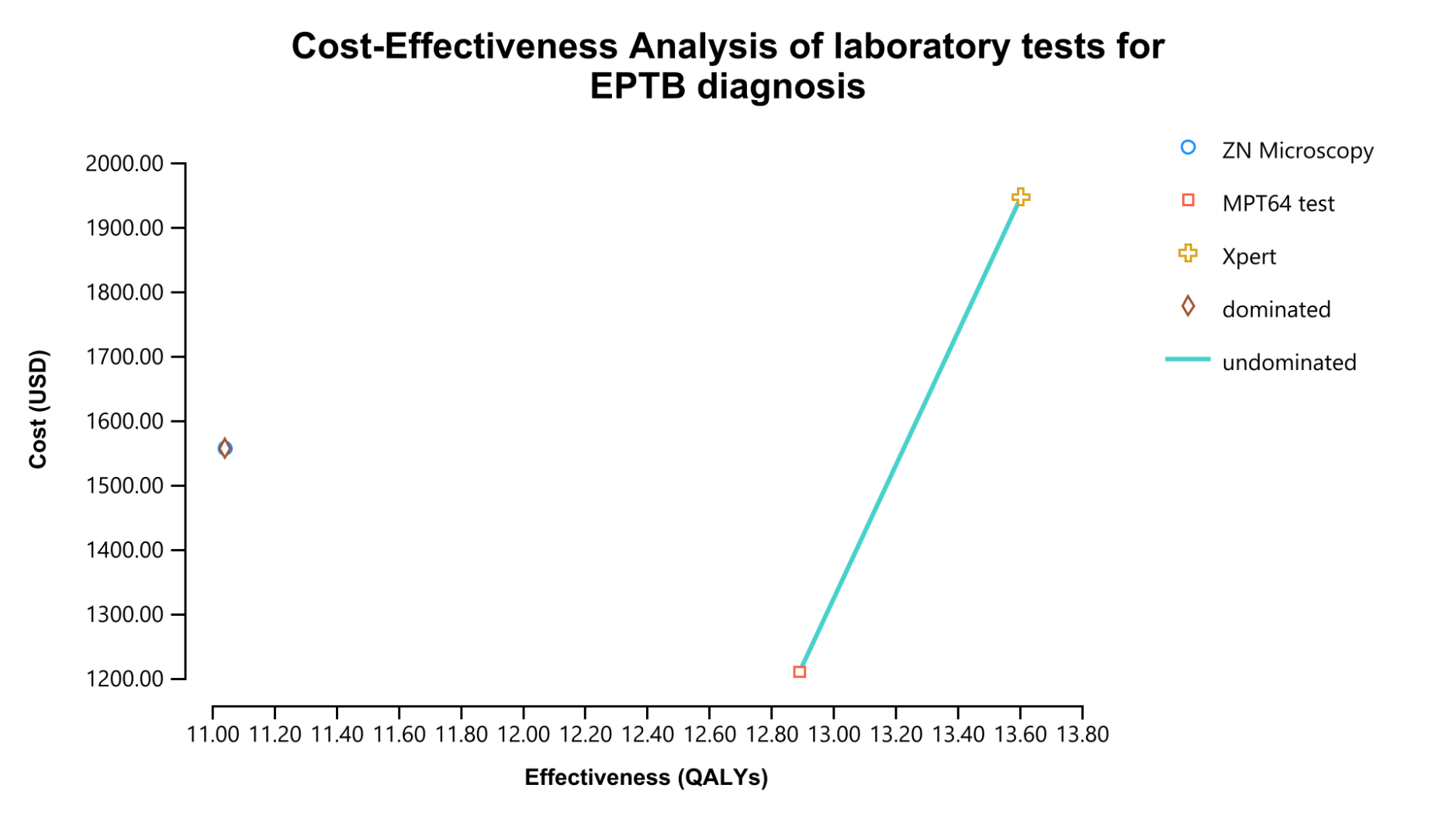


**Fig U. Cost-effectiveness plane showing the Markov model PSA per model input at baseline (tables 1-3), except probability of Xpert test sensitivity at 0.79.**

1. Jørstad MD, Aẞmus J, Marijani M, Sviland L, Mustafa T. Diagnostic delay in extrapulmonary tuberculosis and impact on patient morbidity: A study from Zanzibar. PloS one. 2018;13(9):e0203593-e.

2. EUROQUL. EQ-5D-3L 2022 [Available from: <https://euroqol.org/eq-5d-instruments/eq-5d-3l-about/>.

3. Jelsma J, Hansen K, de Weerdt W, de Cock P, Kind P. How do Zimbabweans value health states? Population Health Metrics. 2003;1(1):11.

4. Agota Szende MOND. EQ-5D Value Sets: Inventory, Comparative Review and User Guide. Monographs EG, editor: Springer Dordrecht; 2010.

5. The World Bank. Inflation, GDP deflator (annual %) 2023 [Available from: <https://data.worldbank.org/indicator/NY.GDP.DEFL.KD.ZG>.

6. Turner HC, Lauer JA, Tran BX, Teerawattananon Y, Jit M. Adjusting for Inflation and Currency Changes Within Health Economic Studies. Value Health. 2019;22(9):1026-32.
